# Microbiome network in the pelagic and benthic offshore systems of the northern Adriatic Sea (Mediterranean Sea)

**DOI:** 10.1038/s41598-022-21182-8

**Published:** 2022-10-05

**Authors:** Daniel Scicchitano, Marco Lo Martire, Giorgia Palladino, Enrico Nanetti, Marco Fabbrini, Antonio Dell’Anno, Simone Rampelli, Cinzia Corinaldesi, Marco Candela

**Affiliations:** 1 Department of Pharmacy and Biotechnology, University of Bologna, Via Belmeloro 6, 40126 Bologna, Italy; 2 Fano Marine Center, The Inter-Institute Center for Research On Marine Biodiversity, Resources and Biotechnologies, Fano, Italy; 3 Department of Materials, Environmental Sciences and Urban Planning, Polytechnic University of Marche, Via Brecce Bianche, 60131 Ancona, Italy

**Keywords:** Next-generation sequencing, Microbial communities, Environmental microbiology

## Abstract

Because of their recognized global importance, there is now the urgent need to map diversity and distribution patterns of marine microbial communities. Even if available studies provided some advances in the understanding the biogeographical patterns of marine microbiomes at the global scale, their degree of plasticity at the local scale it is still underexplored, and functional implications still need to be dissected. In this scenario here we provide a synoptical study on the microbiomes of the water column and surface sediments from 19 sites in a 130 km^2^ area located 13.5 km afar from the coast in the North-Western Adriatic Sea (Italy), providing the finest-scale mapping of marine microbiomes in the Mediterranean Sea. Pelagic and benthic microbiomes in the study area showed sector specific-patterns and distinct assemblage structures, corresponding to specific variations in the microbiome network structure. While maintaining a balanced structure in terms of potential ecosystem services (e.g., hydrocarbon degradation and nutrient cycling), sector-specific patterns of over-abundant modules—and taxa—were defined, with the South sector (the closest to the coast) characterized by microbial groups of terrestrial origins, both in the pelagic and the benthic realms. By the granular assessment of the marine microbiome changes at the local scale, we have been able to describe, to our knowledge at the first time, the integration of terrestrial microorganisms in the marine microbiome networks, as a possible natural process characterizing eutrophic coastal area. This raises the question about the biological threshold for terrestrial microorganisms to be admitted in the marine microbiome networks, without altering the ecological balance.

## Introduction

In marine-ecosystems microbes represent the most abundant and diverse biological components, and account for up to 10% of the total microbial biomass on our planet^1, 2^. Microbes, including bacteria, are responsible for energy fluxes in the marine food webs^3, 4^, and play a central role in the global biogeochemical cycles and ecosystem functioning^5–7^. Because of their global importance, numerous studies have focused on mapping the diversity of marine microbiomes, to understand their distribution patterns and environmental drivers^8–10^ and to assess their potential response to future climate changes^11–13^. So far, contrasting patterns have been reported, potentially due to the different spatial scales, habitat characteristics and the level of taxonomic resolution at which studies have been conducted^8, 14–16^. Relevant insights in this direction have been provided by Tara Ocean in 2015^17^, which collected up to 35.000 samples from surface to mesopelagic waters at the global scale to provide a first inventory of the global diversity of microbiomes and to identify factors shaping their composition. These investigations revealed that longitude and environmental factors (mainly temperature and dissolved oxygen) combine to shape the microbiome composition in the global oceans and are responsible, at least in part, of the observed biogeographical patterns. Conversely, latitude explained only a minimal fraction of the observed diversity^9^. Other key studies based on a systematic and coupled analysis of the pelagic and benthic microbiomes from globally distributed samples, showed structured biogeographical patterns of marine bacterial assemblages, only partially explained by the assessed environmental factors (e.g., temperature, oxygen and pH). Furthermore, a remarkable difference in the composition of pelagic and benthic bacterial assemblages was observed, revealing a pelagic-benthic coupling^18^ limited to the 7% of the total communities in open waters.

Studies, which specifically addressed the latitudinal patterns of diversity at the global scale, reported an increase in microbiome dissimilarity with increasing distance from the sampling points up to 5000 km^17^. However, the diversity of microbiomes at regional scale (distance between sampling sites < 100 km) was only slightly lower than for larger distances, suggesting the existence of a relevant variability in marine microbial communities even at such spatial scale. This finding was also confirmed by studies on the taxonomic composition of benthic prokaryotic assemblages along bathymetric gradients in Mediterranean Sea, which reported high local variability of microbial assemblages^19^, potentially due to intra-specific interactions, limited dispersion, and historical contingencies, which may combine with stochastic physical disturbances^20^. Taken together, these findings suggest the existence of a relevant degree of marine microbiome plasticity at the local scale, both for the pelagic and benthic communities, whose range and degree of variability, as well as functional implications, still need to be dissected. In order to provide some glimpses in this direction, in the present work we conducted a synoptical study on the microbiomes of the water column and surface sediments from 19 sites in a 130 km^2^ area located 13.5 km afar from the Emilia Romagna coast (Italy), in the North-Western Adriatic Sea (Mediterranean Sea).

The Northwestern Adriatic Sea is characterized by shallow waters (maximum depth: ca. 40 m) and, in the coastal area, the ecosystem productivity is mainly sustained by nutrient inputs, especially from the Po river^20,21^. Two currents dominate the circulation in Adriatic: the Western Adriatic Current (WAC), flowing toward the southeast along the Western Italian coast, and the East Adriatic Current (EAC) which flows from the northwest along the eastern Croatian coast^20, 21^.

Riverine inflow into the northern Adriatic forms a buoyant coastal layer—the Western Coastal Layer (WCL)—flowing southward along the Italian coast. The principal compensating inflow occurs along the eastern boundary by EAC, where warm, high-salinity Levantine Intermediate Waters (LIW) is advanced to the North^21, 22^. During the pre-winter and winter periods, after the development of the coastal thermohaline front, the inflow of fresh waters from the Po river (and other sources along the coast) is prevalently retained inside the coastal zone, establishing a dynamic limitation between inshore and offshore systems were riverine nutrients are mainly kept in the coastal area^21, 22^. The spring inversion of the total heat budget leads to a decrease in the density of the surface layer and generates a thermocline. Therefore—during the late spring and summer—the water column is highly stratified^22, 23^ and 3 different layers separates over the whole northern basin. The low-density surface layer is directly influenced by runoff and distribution of diluted riverine waters, while the bottom layer is initially occupied by cold, dense, non-diluted winter waters, later replaced by deep middle Adriatic waters. In these stratified conditions, surface waters flow from the coastal area and inject into the surface layer to reach toward the center of the basin^22–24^.

In our work, by applying 16S rRNA Next Generation Sequencing and network-based approach, we have been able to map the variation at the local-scale of the pelagic and sediment microbiomes in the Northwestern Adriatic Sea. The coupled investigation of the pelagic and benthic microbiomes from each sampling site also allowed us to identify connections, exchanges, and isolation of microbial members in the two realms. Together with the dissection of the respective microbiome network structures, the present study allowed us to provide new insights into the structuring of the marine microbial assemblages at the local and regional scales.

## Materials and methods

### Study area and sampling procedure

**Figure 1:**
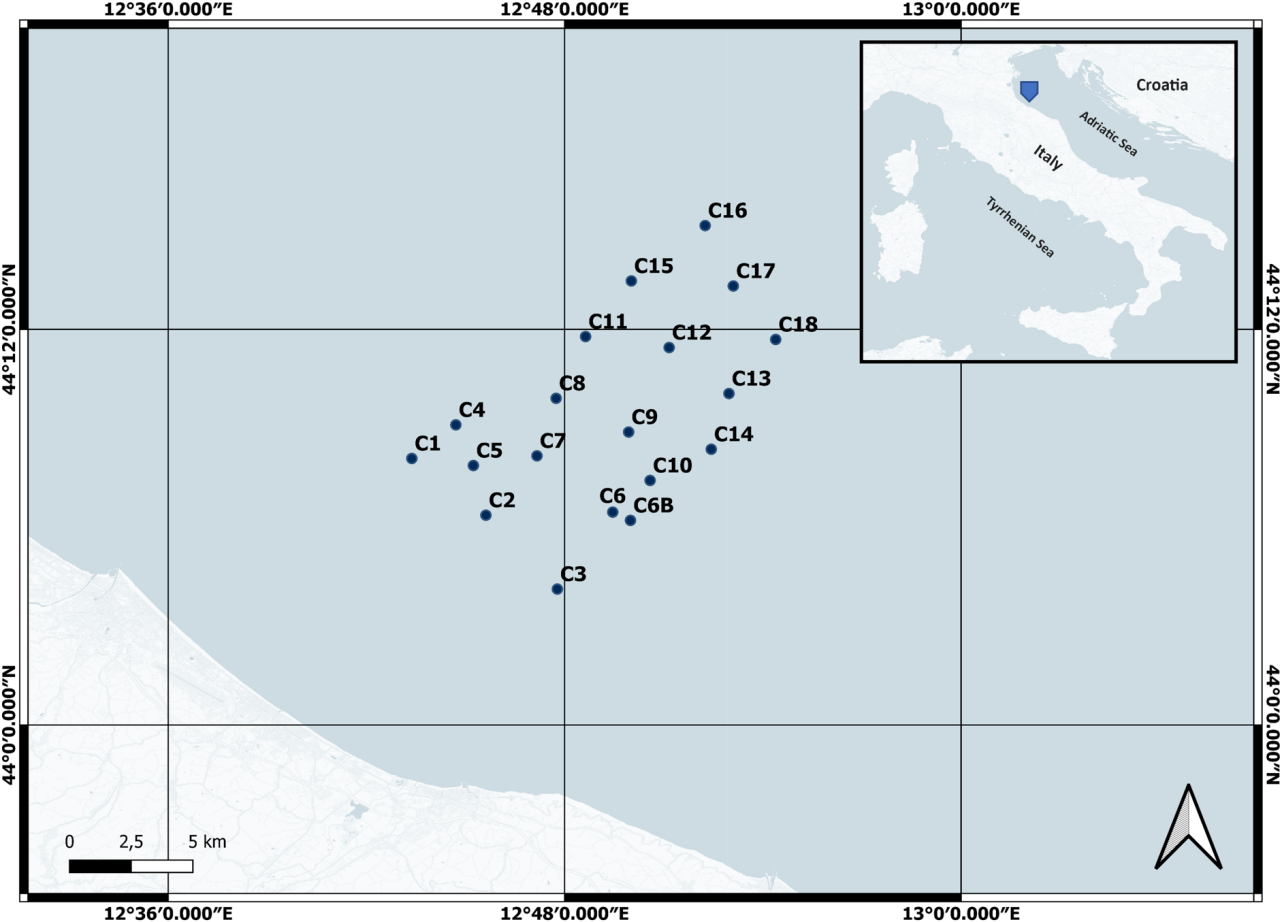
Sampling site and offshore study area. Sampling sites (both for water and sediment) are represented as blue dots.

The present study was conducted in September 2021 in 19 sites (whose geographic coordinates, water depths and distance from coast are reported in Supplementary Table 1) located in an offshore area of 130 Km^2^ in the North-western Adriatic Sea (Latitude: from 44.0686667 to 44.2524444 and Longitude: from 12.72288889 to 12.90647222 M Fig. 1). From each site, one sample of water (10 m depth) and one to 3 samples of sediment were collected, for a total of 19 water samples and 25 sediment samples. Water samples were collected using a Niskin bottle. Immediately after collection, 2L of sea water were poured into a previously sterilized plastic bottle. Surface sediments (the top 10 cm) were also collected, using a Van Veen grab. After homogenization, a portion of 10 g of them was transferred into sterile plastic containers. Samples were stored in the dark until arrival at the laboratory. While sediments were immediately frozen at − 80 °C, water samples were filtered onto 47 mm diameter cellulose mixed ester 0.2 µm pore-size filters (MF-Millipore) through vacuum filtration system^25^ under laminar flow hood. Filters were stored in sterile Eppendorf at − 80 °C until processed.

### Biochemical components of the sedimentary organic matter

Chlorophyll-*a*, phaeopigment, protein, carbohydrate and lipid concentrations in sediment samples were analyzed according to Danovaro^26^. Briefly, chlorophyll-*a* and phaepigments were analyzed fluorometrically and total phytopigment concentrations were defined as their sum. Proteins, carbohydrates and lipids were determined spectrophotometrically^26^. Concentrations of proteins, carbohydrates, and lipids were converted into carbon equivalents using 0.49, 0.40 and 0.75 mgC mg^−1^, as conversion factors, respectively and their sum defined as biopolymeric carbon (BPC, a proxy of available trophic resources,^27^).

### Microbial DNA extraction

Extraction of the total DNA from water samples was performed from the entire membrane filters using the DNAeasy PowerWater extraction kit (QIAGEN, Hilden, Germany) following the manufacturer’s instructions^28^. For sediment samples, 250 mg of each sample was weighed, and total DNA was extracted with the DNAeasy PowerSoil kit (QIAGEN, Hilden, Germany) according to the manufacturer’s instructions with minor adjustments. Specifically, all samples were homogenized using the FastPrep instrument (MP Biomedicals, Irvine, CA) at 5.5 movements/s for 1 min, repeated for three cycles, and the elution step was preceded by a 5-min incubation at 4 °C^29^. Extracted DNA was then quantified by using NanoDrop ND-1000 (Nano Drop Technologies, Wilmington, DE) and stored at − 20 °C until further processing.

### Sequencing, library preparation and bioinformatic analysis

PCR amplification of the V3-V4 hypervariable region of the 16S rRNA gene was carried out in a 50 µL final volume reaction containing 25 ng of microbial DNA, 2X KAPA HiFi HotStart ReadyMix (Roche, Basel, Switzerland), and 200 nmol/L of 341F and 785R primers carrying Illumina overhang sequencing adapter. For water samples the thermal cycle consisted of 3 min at 95 °C, 25 cycles of 30 s at 95 °C—30 s at 55 °C and 30 s at 72 °C, and a final elongation step of 5 min at 72 °C^28^. Sediment samples followed the same PCR amplification protocol with a total of 30 amplification cycles^29^. PCR products were purified with Agencourt AMPure XP magnetic beads (Beckman Coulter, Brea, CA, USA). Indexed libraries were prepared by limited-cycle PCR with Nextera technology and cleaned-up as described above. Libraries were normalized to 4 nM and pooled. The sample pool was denatured with 0.2 N NaOH and diluted to a final concentration of 6 pM with a 20% PhiX control. Sequencing was performed on an Illumina MiSeq platform using a 2 × 250 bp paired-end protocol, according to the manufacturer’s instructions (Illumina, San Diego, CA, USA). A pipeline combining PANDAseq^30^ and QIIME2^31^ was used to process raw sequences. High-quality reads (min/max length = 350/550 bp) were retained using the “fastq filters” function of Usearch11^32^. Specifically, reads with an expected error per base E = 0.03 (i.e., 3 expected errors every 100 bases) were discarded, based on the phred Q score probabilities. The resulting reads from the length and quality filtering were binned into amplicon sequence variants (ASVs) using DADA2^33^, the Taxonomy was assigned using the VSEARCH algorithm^34^ against SILVA database (December 2017 release)^35^. All the sequences assigned to eukaryotes (i.e., chloroplasts and mitochondria) or unassigned were discarded. Sequencing reads were deposited in ENA (project number PRJEB52873).

### Definition of the alpha-diversity sectors

The QGIS software^36^ was used to construct the maps of the study area and to construct the maps based on the Shannon alpha diversity values of each water and sediment sample. The longitude and latitude geographical coordinates (Supplementary Table 1) were used to plot the precise sampling locations into the software. The distribution of the Shannon alpha diversity values across the samples was obtained through the *Triangulated Irregular Network* interpolation method on QGIS (TIN interpolation). In order to define the alpha-diversity sectors, for both the water and the sediment microbiomes, samples distribution according to the Shannon alpha-diversity values were first obtained. The obtained ranks have been than utilized for the identification of correspondent alpha-diversity sectors in the area under study. More specifically, for the water microbiome, the following alpha-diversity sectors have been identified: (1) South-sector, where > than 70% of the correspondent samples were included in the 3° and 4° alpha-diversity quartiles; (2) Central-sector, where 100% of the correspondent samples were included in the 1° and 2° alpha-diversity quartiles; (3) North-sector, where > than 65% of the of the correspondent samples were included in the 3° and 4° alpha-diversity quartiles. Analogously, for the sediment microbiome, the following sectors have been identified: (1) South sector, where > than 80% of the correspondent samples were included in the 1° and 2° alpha-diversity quartiles; (2) North-east sector, where 90% of the correspondent samples were included in the 3° and 4° alpha-diversity quartiles; (3) North-west sector, where > than 80% of the of the correspondent samples were distributed between in the 2° and 4°alpha-diversity quartiles. The quartile distribution of the water and sediment samples and the corresponding sector are reported In Supplementary Table 2.

### Biostatistical analysis and networks construction

All statistical analyses were performed using the R software^37^, using the packages “Made4”^38^ and “vegan”^39^. Unweighted UniFrac distances were plotted using the vegan package, and the data separation in the Principal Coordinates Analysis (PCoA) was tested using a permutation test with pseudo-F ratios (function “adonis” in the vegan package). Wilcoxon rank-sum test and Kruskal–Wallis test were used to assess significant differences in alpha diversity and taxon relative abundance between groups. *P*-values were corrected for multiple testing with “p.adjust” function in R, with a false discovery rate (FDR) ≤ 0.05 considered statistically significant. Bacterial co-abundance groups (CAGs) were identified as previously described^40–42^. Briefly, the associations among the bacterial orders were evaluated using the Kendall correlation test visualized using hierarchical Ward clustering with a Spearman correlation distance metrics. The Wiggum plot network analysis was created using Cytoscape^43^. Circle sizes were proportional to orders abundance or over-abundance, and connections between nodes were represented as “gray line” or “red line” for positive or negative correlation, respectively. Over-abundance values were calculated using the ratio between the mean relative abundance in a specific area and the average relative abundance in the whole area of the study (meanArea/meanTot). Hub nodes, cohesion and modularity identification/calculation were based on area-specific networks obtained by FlashWeave^44^ and the correspondent samples for each area. Specifically, hub nodes were identified for each microbial network by looking to the combination of the highest values of closeness centrality, betweenness centrality and degree on Cytoscape^43^ as previously described^45^. Cohesion and modularity were calculated with the “igraph” package in R following the same procedures proposed by Hernandez and colleagues^46^.

## Results

### Assessment of environmental parameters in the study area

Sampling sites and the studied area are represented in Fig. 1. During the sampling campaign, the temperature of superficial seawater was 23 °C whereas at 10-m depth of 10 °C. Data on the concentrations of proteins (PRT), carbohydrates (CHO) and lipids (LIP) as well as chlorophyll-a (Chl-a), phaeopigments (PHEO) and biopolymeric C (PBP) in the sediment samples are reported in Supplementary Table 3. In the study area, PRT were the dominant class of the investigated organic compounds, ranging from 1.4 to 7.96 mg/g (mean value of 4.18 ± 0.39 mg/g). CHO concentrations varied from 0.27 to 1.24 mg/g (mean value: 0.66 ± 0.06 mg/g), while LIP ranged from 0.25 to 1.62 mg/g (mean value: 0.74 ± 0.07 mg/g). Chl-a and PHEO concentrations in the sediments were, on average, 1.22 ± 0.22 µg/g and 14.66 ± 1.04 µg/g, respectively (range: 0.35–5.37 µg/g for Chl- and 6.49–26.91 µg/g for PHEO). Finally, the range of variability of BPC concentrations was comprised between 0.92 and 5.14 mg/g, with a mean value of 2.86 ± 0.26 mg/g.

### Composition of pelagic and sediments microbiomes

**Figure 2:**
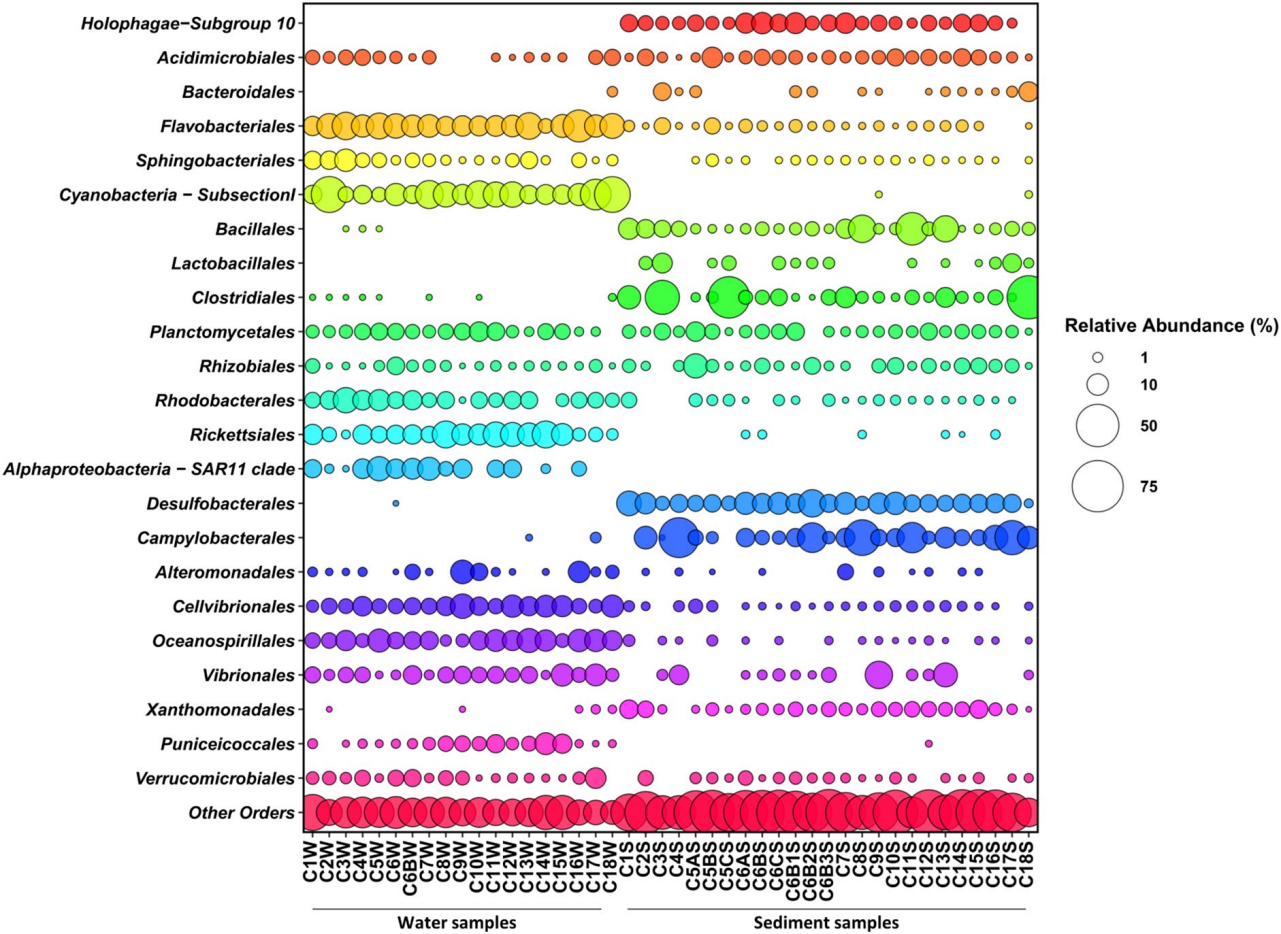
Bubble chart showing the relative abundance of major orders (r.ab. > 5% in at least two samples) in the water samples (left part of the graph) and sediment samples (right part of the graph).

The V3/V4 regions from the 16 s rRNA gene was successfully sequenced from a total of 44 samples (19 waters and 25 sediments), providing 549′318 high quality reads (12′485 ± 3′235 per sample) clustered in 8′271 Amplicon Sequence Variants (ASVs) (206.8 ± 94.4 per sample). None of the detected ASVs have been assigned at the species level, while the assignment rates at the genus, family and order levels were 42, 48 and 48%, respectively. The total diversity at ASVs level was 150 for the water microbiome and 218 for the sediment one. When we assessed for the total assigned diversity at the different phylogenetic ranks, the order level showed the highest value (27.61), respect to family and genus levels scoring 23.8 and 12.96, respectively. In Supplementary Fig. 1 the general compositional structure of the water and sediment microbiomes at the order level is provided. For the pelagic microbiome, the dominant orders were: *Synechoccus-like Cyanobacteria Subsection I* (relative abundance, rel.ab., 13.4%), *Flavobacteriales* (rel. ab. 12.3%), *Rickettsiales* (rel. ab. 8.2%), *Oceanospirillales* (rel. ab 7.1%), *Cellvibrionales* (rel. ab. 6.5%) and *Rhodobacterales* (rel. ab. 5.3%). Among the subdominant fraction, the most represented orders were: *SAR11 clade* (rel. ab. 4.5%), *Vibrionales* (rel. ab. 4.2%), *Planctomycetales* (rel. ab. 3.4%), *Rhodospirillales* (rel. ab. 3.3%), *Sphingobacteriales* (rel. ab 2.9%) and *Verrucomicrobiales* (rel. ab. 2.3%). Differently, sediments were dominated by *Campylobacterales*, *Clostridiales*, *Desulfobacterales*, *Bacillales* and *Holophagae*-*Subgroup 10*, showing relative abundances of 10.3, 7.8, 7.4, 5.0 and 5.4%, respectively. For the benthic microbiome, the most represented minor components were: *Acidimicrobiales* (re. ab. 2.7%), *Planctomycetales* (rel. ab. 2.7%), *Xanthomonadales* (rel. ab. 2.6%), *Rhizobiales* (rel. ab. 2.5%), *Vibrionales* (rel. ab 2.1%), *Holophagae*-*Subgroup 23* (rel. ab 1.5%), *Lactobacillales* (rel. ab 1.3%) and *Flavobacteriales* (rel. ab 1.2%). In order to explore specificities and connections between pelagic and sediment microbiomes, we provide the bubble plots of the distribution of the different microbiome orders in the two ecosystems (Fig. 2). According to our findings, *Synechoccus-like Cyanobacteria Subsection I, SAR11 clade* and *Puniceicoccales* were characteristic of the pelagic microbiome, while *Holophagae-Subgroup 10*, *Bacteroidales*, *Bacillales*, *Lactobacillales*, *Clostridiales*, *Desulfobacterales* and *Campylobacterales* and *Xanthomonadales* were almost exclusive for the sediment one. However, several orders were shared between the two ecosystems. In particular, *Flavobacteriales*, *Rickettsiales*, *Cellvibrionales*, *Oceanospirillales*, *Alteromonadales, Rhodobacteriales,* and *Vibrionales* were most abundant in water samples, and *Verrucomicrobiales*, *Rhizobiales*, *Planctomycetales* and *Acidomicrobiales* were almost equally represented in water and sediment samples.

### Changes in abundance and diversity of the pelagic and sediment microbiomes at the local scale

**Figure 3:**
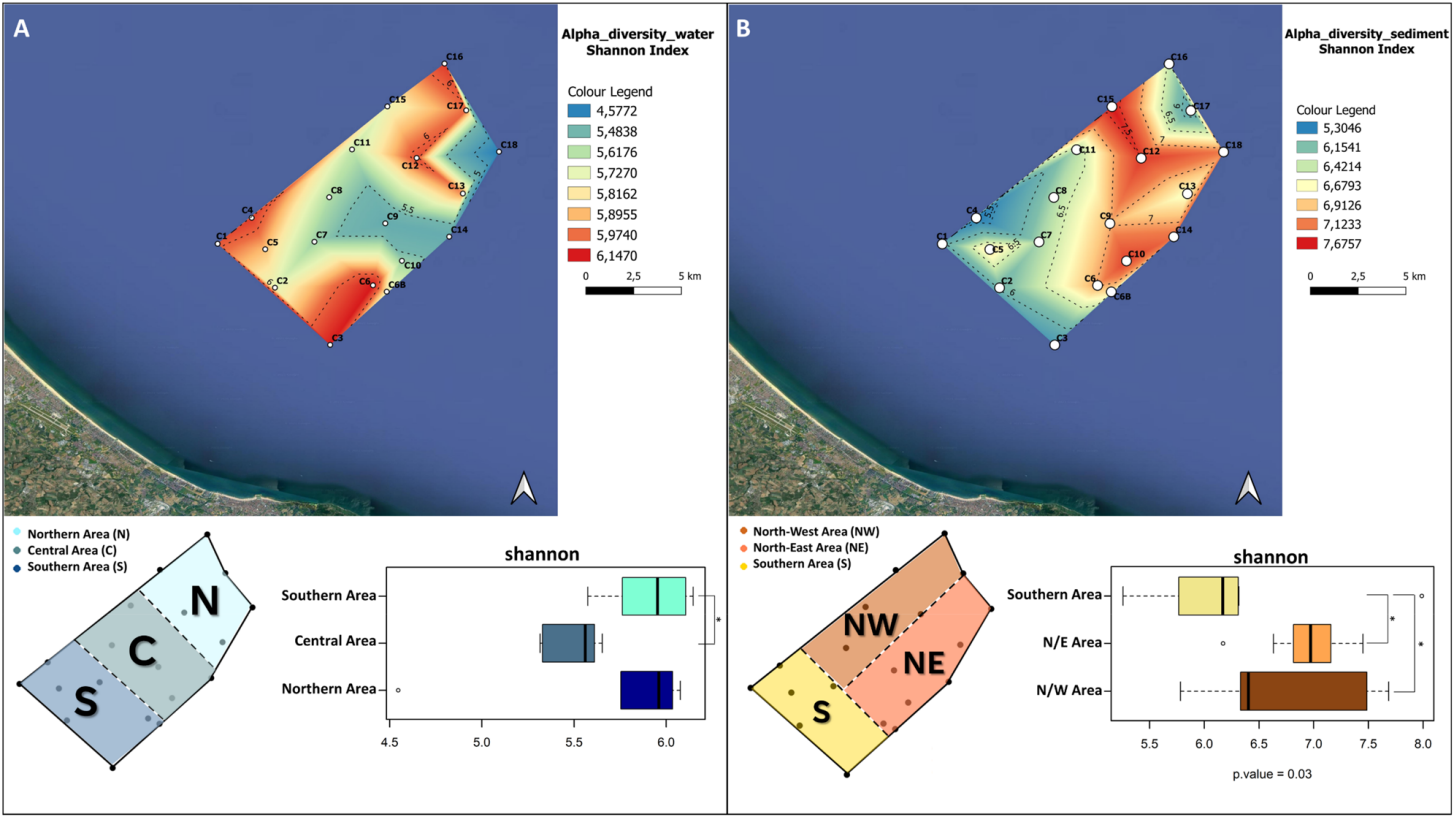
Representation of the alpha-diversity patterns in the area for both water and sediment samples. (**A**) (Upper part) Distribution of the alpha diversity values among water samples, generated with TIN interpolation of the single values. Colour scale from red to blue represents a decrease in alpha diversity; black lines represent contour lines of the interpolation. (Bottom part) Subset of samples divided into 3 areas based on Shannon diversity; for water samples, the Northern Area, the Central Area and the Southern Area were identified. Box plot of Shannon index calculated for the 3 identified areas of water samples. (**B**) (Upper part) Distribution of the alpha diversity values among sediment samples, generated with TIN interpolation of the single values. Colour scale from red to blue represents a decrease in alpha diversity; black lines represent contour lines of the interpolation. (Bottom part) Subset of samples divided into 3 areas based on Shannon diversity; for sediment samples, the North-West Area, the North-East Area and the Southern Area were identified. Box plot of Shannon index calculated for the 3 identified areas of sediment samples (Wilcoxon rank-sum test; *p* < 0.05*).

**Figure 4:**
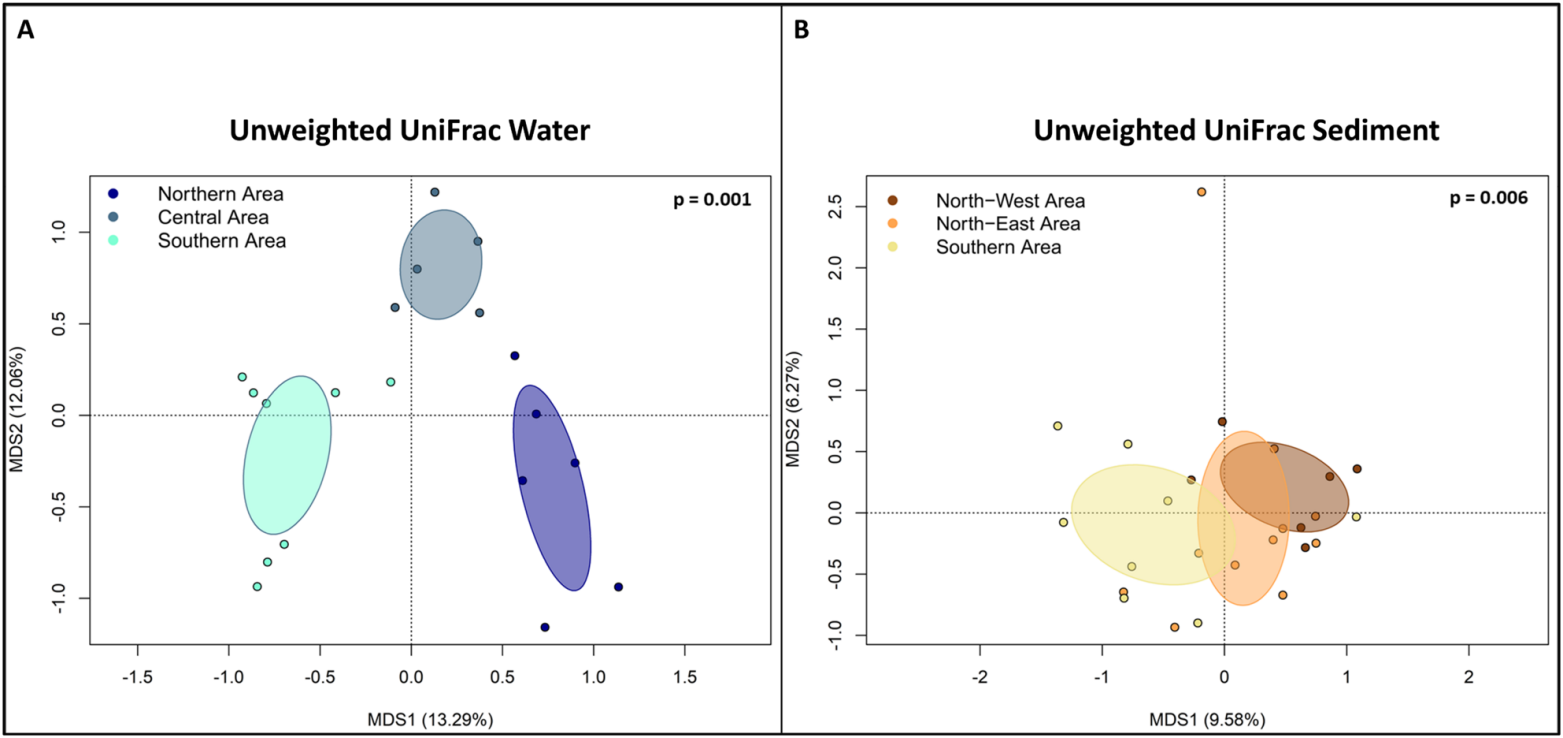
Beta diversity of the bacterial community of the water (**A**) and sediment (**B**) samples in the studied area. (**A**) PCoA based on unweighted UniFrac distances between pelagic microbiome of the 3 areas, samples are significantly separated (Adonis; *p* = 0.001). (**B**) PCoA based on unweighted UniFrac distances between sediment microbiome of the 3 areas, samples are significantly separated (Adonis; *p* = 0.006).

**Figure 5:**
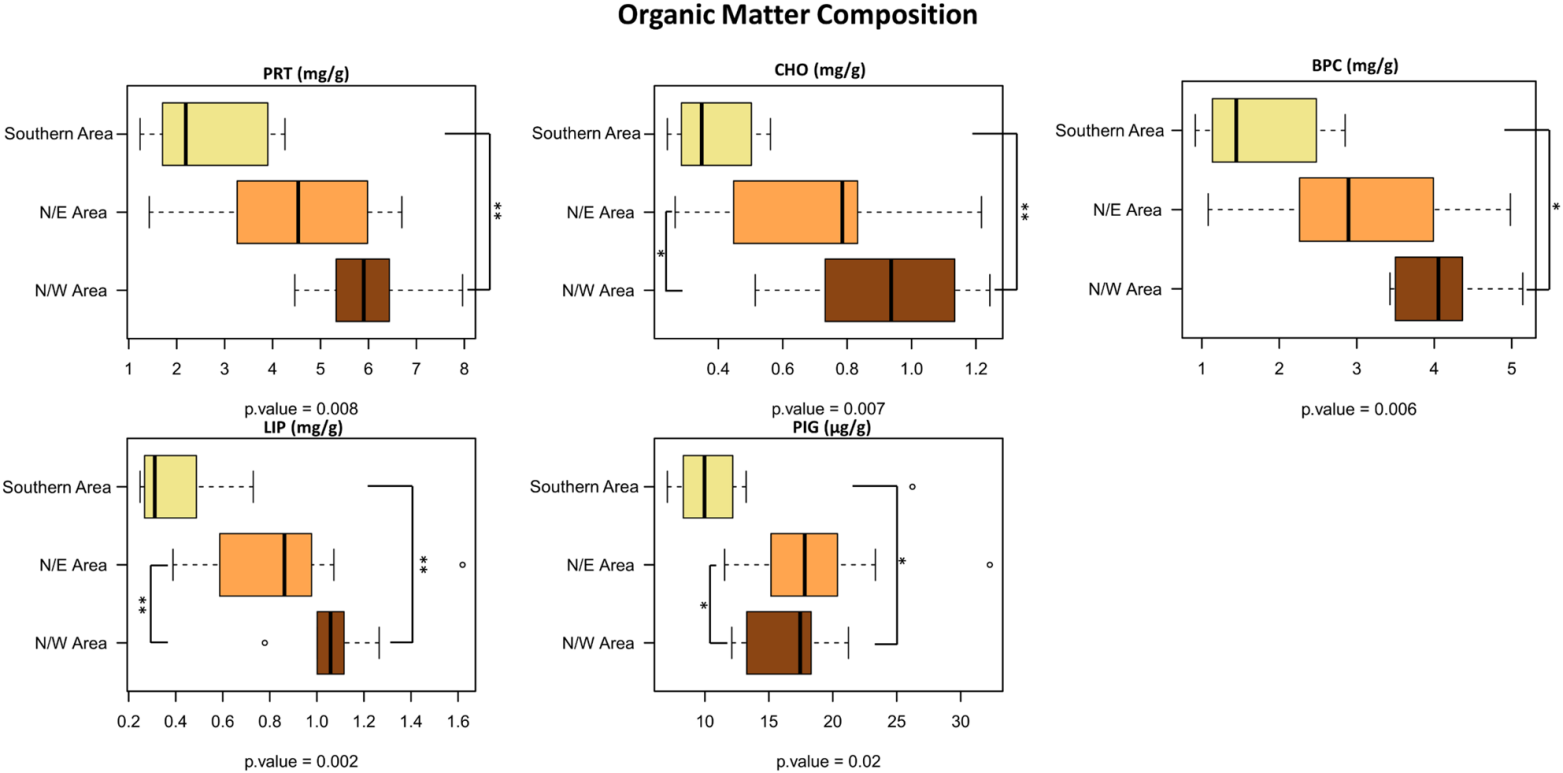
Boxplots showing the variation of the biochemical components across the 3 sectors of the area under study, in terms of concentration (mg/g or µg/g). The central box of each dataset represents the distance between the 25th and the 75th percentiles. The median between them is marked with a black line. Significant variations across groups are highlighted in the figure (Wilcoxon rank-sum test; *p* ≤ 0.05*, *p* ≤ 0.01**). PRT: Total Proteins; CHO: Total Carbohydrates; LIP: Total Lipids; PIG: Total Phytopigments; BPC: Biopolymeric Carbon.

To identify change in the microbiome patterns in the study area, we first accounted for changes in alpha and beta-diversity across the 3 transects. Specifically, to highlight alpha-diversity patterns, the area plots of the Shannon index variation in water and sediments samples were computed (Fig. 3). For both the pelagic and sediment microbiomes high and low alpha-diversity sectors have been identified, showing significant differences in Shannon diversity values. For the pelagic ecosystem, the North and the South sectors were characterized by microbiomes with higher alpha-diversity, compared with the Central sector (Fig. 3A). Similarly, for the sediment microbiome, two high alfa-diversity sectors were identified in the North-East and North-West sectors, while a lower diversity area was detected in the Southern sector (Fig. 3B). We subsequently assessed beta-diversity patterns in the study area. To this aim, the PCoA of the ASVs variation in water and sediment microbiome samples was carried out. According to our findings, for the pelagic microbiome, samples belonging to the previously identified alpha-diversity sectors—South, Central and North—significantly segregated in the PCoA plot (Adonis; *p* = 0.001; Fig. 4A**)**. Similarly, for the sediment microbiome, samples segregated according to the corresponding alpha-diversity sectors (North-Est, North-West and South) (Adonis, *p* = 0.006) (Fig. 4B). When we searched for the correlations between PCoA coordinates and water column depth or distance from the coast, significant relationships were detected for both pelagic and sediment microbiomes (Supplementary Figure S2). For the pelagic microbiomes, the MDS1 significantly correlated with depth (R = 0.9, *p* < 0.005) and distance from the coast (R = 0.9, *p* < 0.005), while, for the sediment microbiome, we obtained analogous significant correlations but with MDS2, R = 0.25, *p* < 0.01. Further, when we assessed correlations among samples alpha-diversity and PCoA coordinates, a positive correlation with MDS1 was observed for sediment microbiomes (R = 0.6, *p* < 0.005), while only tendencies were obtained for the pelagic microbiome. When we accounted for differences in the biochemical composition in sediments corresponding to the sectors, we observed a higher concentration of all biochemical components of sedimentary organic matter (proteins, carbohydrates, lipids and total phytopigments) in the Northern sectors (Fig. 5**)**. Finally, the correlation between the UniFrac distances matrix of sediment microbiome samples and the correspondent distance matrices of the biochemical composition was significant (Table 1**)** (Mantel Test in R). When we assessed the linear regression between the different microbial orders detected in the sediment microbiome and the concentrations of LIP, PRT and CHO, no biological relevant correlations were detected (R^2^ > 0.25) (Supplementary Figure S3).

**Table 1:** Output of Mantel test analyses on the Spearman correlation of the unweighted UniFrac distances of microbiome structure and distance matrix of biochemical components generated with *dist* function in R (method = “*Euclidean*”), number of permutations: 9999.

	Mantel statistic r	Significance (*p* value)
Carbohydrates x UniFrac distances	0.2552	2.7e-03
Proteins correlation x UniFrac distances	0.2701	6e-04
Lipids correlation x UniFrac distances	0.3118	7e-04
Phytopigments correlation x UniFrac distances	0.3275	1.6e-3

### Variation in the pelagic and sediment microbiomes network structure at the local scale

**Figure 6:**
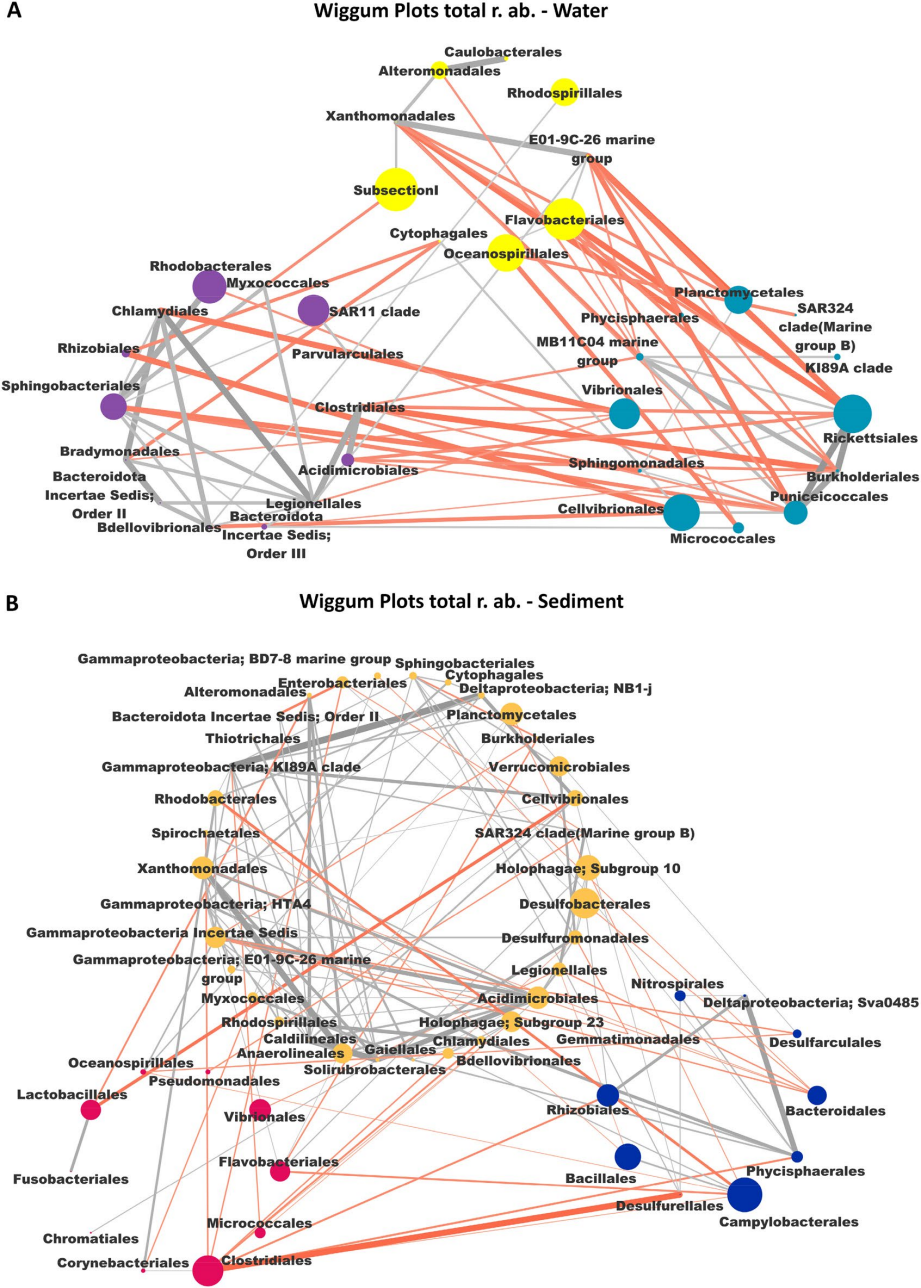
Wiggum plots representing the overall relative abundance of each bacterial order in the 3 CAGs for water (**A**) and sediment (**B**) microbiome. CAGs are named according to the most abundant order and are colour coded as follows: (**A**) *Rhodobacterales* (Violet), *Vibrionales* (Bondi Blue) and *Flavobacteriales* (Yellow) for water microbiome; (**B**) *Desulfobacterales* (Ocher), *Clostridiales* (Pink) and *Campylobacterales* (Blue) for sediment microbiome. Each node represents a bacterial order, and its dimension is proportional to its mean relative abundance in all samples. Connections between nodes represent positive (gray) and negative correlation (red) between order.

With the attempt to better identify the community-level implications of diversity patterns observed for the pelagic and sediments microbiomes in the study area, a network-based approach was applied. To this aim, the overall network structure of the pelagic and sediment microbiomes was obtained and then the correspondent declinations in the different sectors were assessed. For the creation of the overall microbiome networks, the co-abundance associations between orders were computed, then orders were clustered in co-abundance groups CAGs (Supplementary Figure S4)*.* For both ecosystems, 3 different CAGs were detected and named according to the dominant order. The CAGs composition is provided in the Supplementary Table 4. For the pelagic microbiome, the detected CAGs were *Rhodobacteriales* CAG, the *Vibrionales* CAG and the *Falavobacteriales* CAG, while for the sediment microbiomes the correspondent CAGs were *Desulfobacterales* CAG, *Clostridiales* CAG and *Campylobacterales* CAG. In Fig. 6, we provided the Wiggum plots of the overall network structure of the pelagic and sediment microbiomes, where the compositional relationships between the correspondent CAGs are represented.

**Figure 7:**
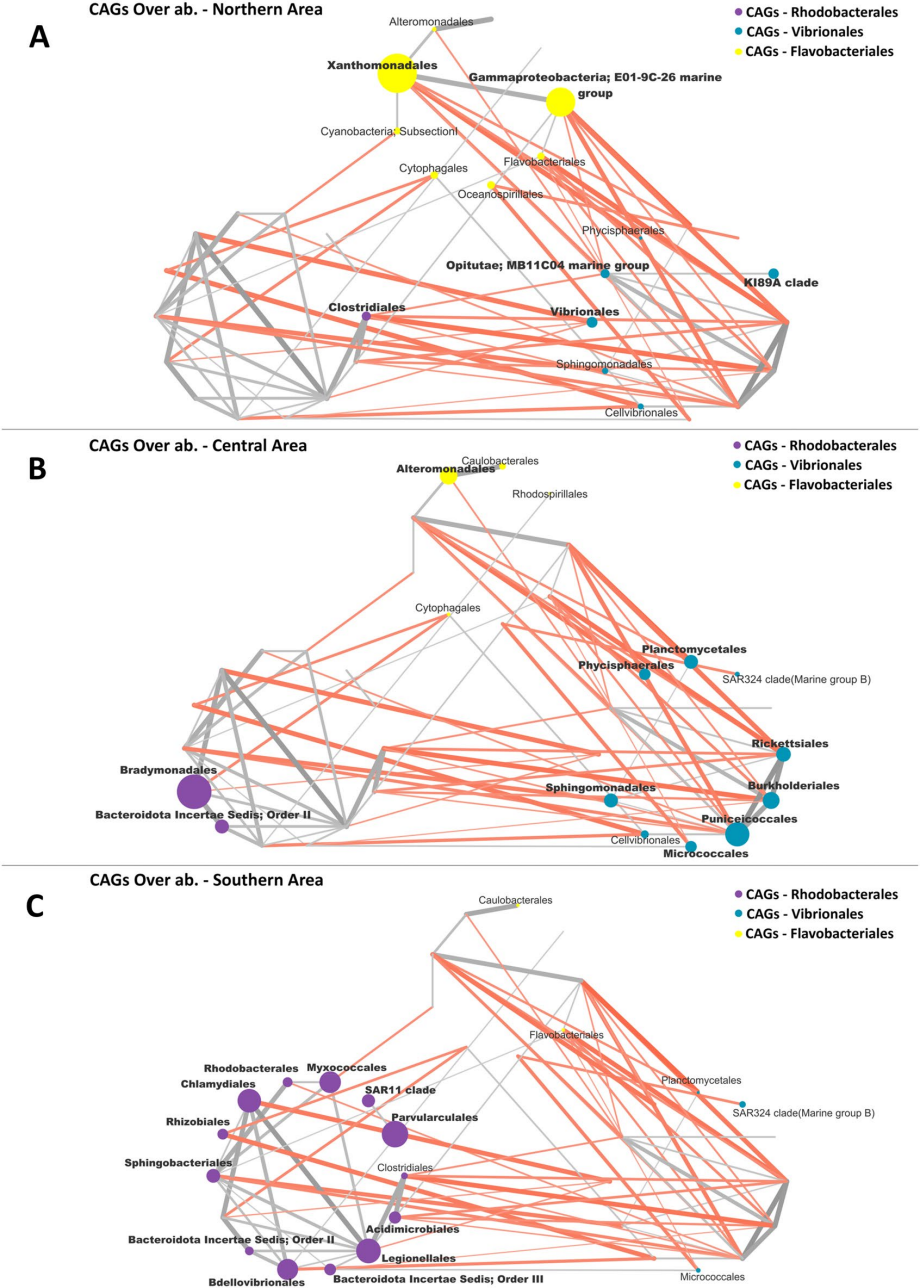
Wiggum plots representing the over-abundance of each bacterial order in the 3 CAGs in the water ecosystem in each Area: (**A**) Northern Area; (**B**) Central Area and (**C**) Southern Area. CAGs are named according to the most abundant order in each CAG and are colour coded as follows: *Rhodobacterales* (Violet), *Vibrionales* (Bondi Blue) and *Flavobacteriales* (Yellow). Each bacterial order is depicted as a node whose size is proportional to its over-abundance. Node and name of bacterial order with an over-abundance < 1 are not represented, and those with an over-abundance ≥ 1.3 are bolded.

**Figure 8:**
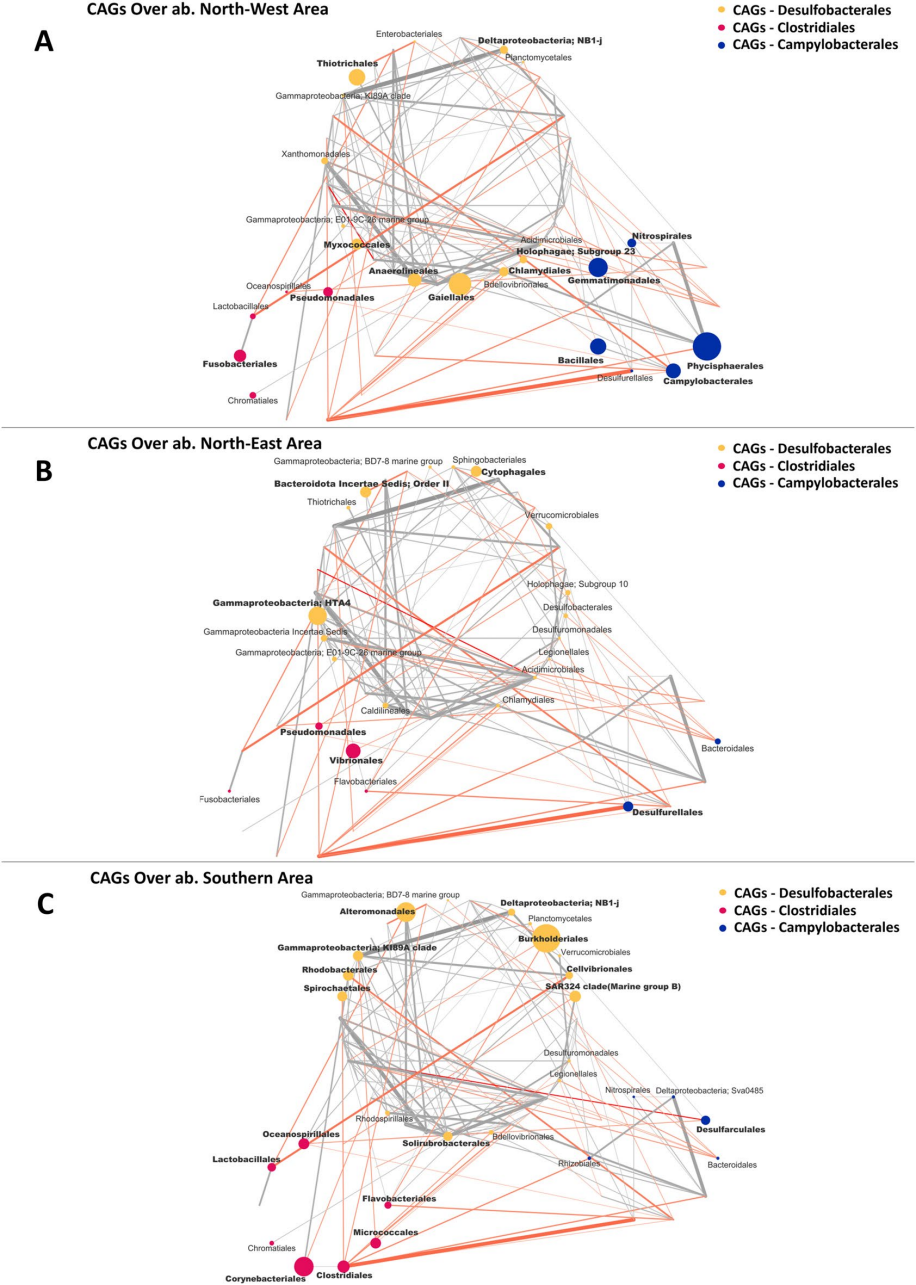
Wiggum plots representing the over-abundance of each bacterial order in the 3 CAGs in the sediment ecosystem in each Area: (**A**) North-West Area; (**B**) North-East Area and (**C**) Southern Area. CAGs are named according to the most abundant order in each CAG and are colour coded as follows: *Desulfobacterales* (Ocher), *Clostridiales* (Pink) and *Campylobacterales* (Blue). Each bacterial order is depicted as a node whose size is proportional to its over-abundance. Node and name of a bacterial order with an over-abundance < 1 are not represented, and those with an over-abundance ≥ 1.3 are bolded.

The variation of the pelagic and sediment networks in the different sectors were than explored. To this aim, for both ecosystems, the sector specific patterns of over-abundance modules (CAGs) and nodes (orders) were computed, and the respective over-abundant network plots were created (Figs. 7 and 8). The box plot showing the variation in relative abundance of the over-abundant CAGs and orders in each sector are provided in Supplementary Figure S5.

According to our findings, for the pelagic microbiomes, the 3 sectors showed a specific pattern of over-abundance CAGs. Particularly, the *Falvobacteriales* CAG was most abundant in the North sector, while the *Vibrionales* CAG and *Rhodobacteriales* CAG were most represented in the Central and South area, respectively. Focusing on the single orders, each sector showed a specific set of over-abundant components: (1) for the North sector: *Xanthomonadales* and *E01-9C-26 marine group* (for the *Flavobacteriales* CAG), *MB11C04 marine group*, *Vibrionales* and *KI89A clade* (for the *Vibrionales* CAG) and *Clostridiales* (for the *Rhodobacteriales* CAG) resulted over-abundant; (2) for the Center sector the over-abundant orders were *Alteromonadales* (for the *Flavobacteriales* CAG), *Planctomycetales*, *Phycisphaerales*, *Sphingomonadales*, *Micrococcales*, *Puniceicoccales*, *Burkholderiales* and *Rickettsiales* (for the *Vibrionales* CAG), *Bradymonadales* and *Bacteroidota Order* II (for the *Rhodobacteriales* CAG); (3) for the South sector were over-abundant the following orders all belonging to the *Rhodobacteriales* CAG: *Chlamydiales*, *Rhizobiales*, *Sphingobacteriales*, *Bacteroidota Orders II and III*, *Bdellovibrionales*, *Legionellales*, *Acidimicrobiales*, *Parvularculales*, *SAR11 clade*, *Myxococcales* and *Rhodobacterales*. For what concern the sediment microbiome, an analogous situation was observed. In particular, at the CAGs level, the North-West sector was enriched in the *Campylobateriales* CAGs, while depleted in *Desulfobacterales* CAGs and *Clostridiales* CAGs compared to North-East and South sectors. For what concerns the orders, the following site-specific over-abundant pattern was observed: (1) *NB1-j*, *Thiotrichales*, *Myxococcales*, *Anaerolineales*, *Gaiellales*, *Chlamydiales*, *Holophagae Subgroup 23* (for the *Desulfobacterales* CAGs), *Pseudomonadales* and *Fusobacteriales* (for the *Clostridiales* CAGs) and *Nitrospirales*, *Gemmatimonadales*, *Bacillales*, *Campylobacterales*, *Phycisphaerales* (for the *Campylobateriales* CAGs) were over-abundant in the North West sector; (2) *Bacteroidota Order II*, *Cytophagales* , *HTA4* (for the *Desulfobacterales* CAGs), *Pseudomonadales Vibrionales* (for the *Clostridiales* CAGs) and *Desulfurellales* (for the *Campylobateriales* CAGs) were over abundant in the North East sector; (3) *Alteromonadales*, *KI89A clade*, *Rhodobacterales*, *Spirochaetales*, *Solirubrobacterales*, *SAR324 clade(Marine group B)*, *Cellvibrionales*, *Burkholderiales* and *NB1-j* (for the *Desulfobacterales* CAG), *Oceanospirillales*, *Lactobacillales*, *Corynebacteriales*, *Clostridiales*, *Micrococcales*, *Flavobacteriales* (for the *Clostridiales* CAGs) and *Desulfarculales (*for the *Campylobateriales* CAGs) were over abundant in the South sector. Finally, for both the water and sediment microbiome, site-specific community networks were created for each of the 3 alpha diversity sectors. For each local network, correspondent key parameters in term of modularity, total connectivity, negative to positive cohesions and hubs orders are provided in Table 2.

**Table 2:** The table represents the parameters of Networks of the single Area, in terms of negative to positive cohesion ratio (N:P), Modules, Total connectivity and hubbs order of the Network, both for pelagic microbiome (first three rows of the table) and sediment microbiome (last three rows of the table).

Single networks parameters					
	N:P Cohesion ratio	Modules	Total connectivity	Hubbs species	
Northern area—Water	1.39	22	1.06	Sphingobacteriales	Cytophagales
Central area—Water	1.63	25	0.69	Planctomycetales	
Southern area—Water	0.39	20	0.77	Acidomicrobiales	Rhizobiales
N/W Area—Sediment	0.95	25	0.58	Rhizobiales	
N/E Area—Sediment	1.5	39	0.71	Planctomycetales	
Southern area—Sediment	0.88	26	0.80	Micrococcales	Desulfobacterales

## Discussion

In the present study we conducted a synoptical analysis of the assemblage composition of the pelagic and sediment microbiomes in a 130 km^2^ offshore area of the Northern-western Adriatic Sea (Mediterranean Sea). The protein contents and proteins to carbohydrates ratio (as a proxy of the nutritional quality of the organic matter) detected in sediment samples allowed to rank the area under study from meso-oligotrophic to eutrophic^47^, generally showing a higher concentration for all the assessed biochemical components with respect what reported in other studies from same geographical area^48^ or other coastal benthic ecosystems worldwide^27^.

According to our findings, the pelagic ecosystem of the investigated area was dominated by *Synechoccus-like Cyanobacteria Subsection I*, a photosynthetic primary producer characteristic of nutrient-rich coastal ecosystems^48, 49^, and by *Flavobacteriales*, *Oceanospirillales* and *Rhodobacteriales*. These latter microorganisms represent aerobic heterotrophs with an important role in the degradation of the dissolved organic matter (DOM) pool, known to prosper as r-strategist in copiotrophic environments such as the Adriatic Sea^50^. Conversely, *SAR11 clade* and *Cellvibrionales*, were only represented as minor components in our samples, being k-strategist cosmopolitan marine heterotrophs which typically dominate oligotrophic waters^51^. Primary producer bacteria and organic carbon degraders were complemented with members involved in sulfur cycling such as *Rhodobacteriales* and *Rhodospirillales,* suggesting a certain balance in nutrient cycling in the pelagic ecosystem of the North Adriatic^52, 53^. Finally, in the pelagic microbiome we detected *Rickettsiales*, as a dominant component, that generally is a host-associated microorganism present in nutrient-enriched ecosystems^54–56^.

For what concerns the sediment microbiome, it was largely dominated by organic carbon fermenters—even with known possible terrestrial origins—such as members of *Clostridiales*, *Bacillales*, *Vibrionales* and *Lactobacillales*^57^. In particular, these microorganisms are known for their importance in the degradation of the organic carbon in anaerobic eutrophic sediments of coastal ecosystems^7, 58, 59^. Furthermore, the sediment microbiome was dominated by microbial components able to reduce sulfate (*Desulfobacterales)* and nitrite (*Acidomicrobiales*) in anaerobic conditions, with an important role in biogeochemical cycling^7, 58, 60^. These components also include *Planctomycetales*, as anaerobes able to perform Anammox^61^. The synoptical investigation of microbiomes in seawaters and sediments allowed us to explore their connections between the two ecosystems. As typical for shallow waters^9^ different microbiome components were shared between pelagic and sediments assemblages—indicating a benthic-pelagic coupling. The shared groups included copiotrophic microorganisms assimilating DOM at low O_2_ levels, such as *Oceanospirillales*, *Alteromonadales*, *Vibrionales*, *Planctomycetales* and *Verrucomicrobiales*^62^ and the anoxygenic phototroph *Rhodobacterales*, known to inhabit shallow sediments^63^.

For both the pelagic and sediment microbiomes, the corresponding networks structures were obtained, allowing for dissecting modules of co-occurring orders as CAGs. Each CAG showed a specific pattern of functional propensity. Particularly, for the pelagic microbiome, the *Flavobacteriales* CAG was characterized by oxygenic phototrophs (*Synechoccus-like Cyanobacteria Subsection I*), DOM assimilating aerobes (*Oceanospirillales* and *Flavobacteriales*), and sulfide oxidizers (*Rhodospirillales*). Differently, the *Vibrionales* CAG was dominated by copiotrophic (*Vibrionales* and *Planctomycetales*) and oligotrophic (*Cellvibrionales* and *K189A* clade) heterotrophs, with the host-associated marine groups *Rickettsiales* as other major components. Marine microorganisms known for their capacity to degrade mono and polycyclic aromatic compounds (*Sphingomonadales* and *Burkholderiales*) were characteristic components of this CAG^64, 65^. Finally, the *Rhodobacteriales* CAG was dominated by host-associated microbial groups, including components of the rhizosphere and plant microbiomes *Sphingobacteriales* and *Rhizobiales*^66, 67^, and predatory microorganisms such as *Bradymonadales, Bdellovibrionales* and *Myxococcales*^68–71^. Important members of this CAG were also *SAR11* bacteria, which are among the most abundant carbon-oxidizing bacteria in pelagic systems^72^.

For the sediment microbiome, the *Desulfobacterales* CAG was characterized by anaerobes involved in N and S cycling, such as *Planctomycetales* and *Acidomicrobiales* (N reducers) and *Desulfobacterales* (S reducers)^7, 58, 60^. Conversely, the *Campylobacterales* CAG was characterized by several anaerobic heterotrophs such as *Bacteroidales, Bacillales* and *Campylobacterales,* the latter being shown to increase in nutrient-enriched waters during microalgal blooms^73^. Finally, the *Clostridiales* CAG was also characterized by carbon fermenters thriving in carbon-rich trophic sediments, but including microorganisms with known terrestrial origin, such as *Clostridiales* and *Lactobacillales*^7, 58, 59^. The hydrodynamic and trophic conditions in the investigated period may explain the largely heterotrophic nature of the pelagic and sediment microbiomes observed in the study area. Indeed, during the summer period, when waters are highly stratified, the limited river inputs (generally characterized by low phytoplankton biomass) reach the offshore systems, where microbial-mediated degradation of organic matter prevails on primary production processes^74^.

Based on our analyses we observed that the pelagic and benthic microbiomes in the study area showed sector-specific patterns and distinct assemblage structures. In particular, the pelagic microbiome was characterized by three compositional clusters corresponding to the South, Central, and North sectors, the second characterized by the lowest alpha-diversity. Analogously, for the sediment microbiome, 3 different configurations were observed, corresponding to the North-eastern, North-western, and the South sectors, the latter showing the lowest alpha-diversity. Interestingly, this observed heterogenicity of the pelagic and sediment microbiomes at the local scale corresponded to detectable variations in the respective microbiome networks. Indeed, sector-specific patterns of over-abundance modules (CAGs) and nodes (orders) were defined. The pelagic microbiome, in the North and the Central sectors, was characterized by the over-abundance of heterotrophic groups belonging to the *Flavobacteriales* and *Vibrionales* CAGs, such as *Flavobacteriales*, *KI89A clad*e, *MB11C04 marine group*, *Clostridiales* and *Vibrionales*, capable to prosper in nutrient-rich waters assimilating DOM^57, 75–77^. The central sector was also characterized by microbial groups known as hydrocarbon degraders, such as *Sphingomonadales* and *Burkholderiales*^64, 65^. Conversely, the over abundant nodes in the South sector mainly belonged to host-associated microbes of possible terrestrial origins (*Chlamydiales*, *Rhizobiales* and *Legionellales*^67, 78, 79^ and predatory orders (*Myxococcales* and *Bdellovibrionales*)^67, 68^, which contributed to the *Rhodobacteriales* CAG, also including marine heterotroph prospering in oligotrophic waters as K strategists as *SAR11 clade*^57^.

Both the North (North-eastern and -western) and the South sites of the sediment microbiome were characterized by copiotrophic carbon fermenters, but possibly showing different origins. Indeed, while fermenters from the North sites mainly belonged to marine heterotrophs such as *Thiotrichales*, *Gaiellales*, *Pseudomonadales*, *Bacillales*, *Campylobacterales*, *Phycisphaerales* and *Vibrionales*^53, 80–83^, in the South area fermenters belonged to microbial orders of possible terrestrial origin, such as *Corynebacteriales*, *Clostridiales*, *Lactobacillales* and *Spirochaetales*. However, despite the heterogenicity in terms of network over-abundant orders at the different sectors identified in the pelagic and benthic systems of the investigated area, important functional categories, such as organic carbon degraders, nitrogen cyclers, sulfur cyclers and, for the pelagic microbiome, carbon fixing microorganism, were always represented, supporting the well-balanced structures of the observed microbiome networks in term of potentiality for global cycling in a copiotroph coastal marine ecosystem.

According to our findings, the concentrations of biochemical components of the sedimentary organic matter in the three sectors were different, with higher values in the Northern Sector. The differences in trophic availability observed between Northern and Southern sectors may explain—at least in part—the different compositional structures of the corresponding sediment microbiome, as shown by the correlation of the correspondent samples distance matrices. These findings support the importance of organic matter as a key driver of microbiome diversity in benthic marine ecosystems^84^. At the same time, the higher relevance of terrestrial microorganisms in the south sectors can be explained by the peculiar hydrodynamic conditions of the Northwestern Adriatic Sea during the summer season when the plume of the Po and other local rivers are mainly transported eastwards, toward the center of the basin, rather than being exported southwards as occur in the winter^22–24^.

## Conclusions

Our findings provide new insights into the local changes of the pelagic and sediments microbiomes in an offshore area of the North-western Adriatic Sea. Based on our results, despite the pelagic and benthic microbial assemblages showed a certain heterogenicity in the investigated area they maintained a well-balanced structure, being always structured for the provision of key ecosystem services (e.g., primary production, nutrient cycling, hydrocarbon degradation). Interestingly, Microbiomes at the different sites showed comparable ecological roles but a different origin, such as those of the South site (i.e., the closest to the coast) where both the pelagic and benthic ecosystems were characterized by microbial groups of terrestrial origin. Interestingly, these terrestrial microorganisms seem to become integral to the marine microbiome networks, as indicated by the comparable degree of modularity and connectivity of the local network at the South sites with respect to the other subarea^42^. Even if our study has a limited phylogenetic resolution and does not allow us to assess temporal microbiome changes, our findings rise possible concerns about the biological threshold, in terms of relative abundance, for terrestrial microorganisms—including the ones of fecal origin—to be included in the marine microbiome networks, without altering the ecological balance. However, in this perspective, more research is needed, with an improved phylogenetic resolution, also expanding the observation to other geographical sites and assessing for seasonal changes.

## Supplementary Information

Supplementary Information.

## Data Availability

The datasets generated and/or analysed during the current study are available in the ENA repository (project number PRJEB52873). Direct link: https://www.ebi.ac.uk/ena/browser/view/PRJEB52873?show=reads.
