# Supplementary material for: Microbiome network in the pelagic and benthic offshore systems of the northern Adriatic Sea (Mediterranean Sea)
Source: Sci Rep. 2022 Oct 5;12:16670. doi: 10.1038/s41598-022-21182-8 (PMC9535000; doi:10.1038/s41598-022-21182-8)
Supplement: Supplementary file 1 — Supplementary Information. [file 41598_2022_21182_MOESM1_ESM.docx]

**Microbiome network in the pelagic and benthic offshore systems of the northern Adriatic Sea (Mediterranean Sea)**

Daniel Scicchitano^1,2^, Marco Lo Martire^3^, Giorgia Palladino^1^, Enrico Nanetti^1^, Marco Fabbrini^1^, Antonio Dell’Anno^3^, Simone Rampelli^1^, Cinzia Corinaldesi^3^, Marco Candela^1,2,*^

^1^Department of Pharmacy and Biotechnology, University of Bologna, Via Belmeloro 6, 40126 Bologna, Italy

^2^Fano Marine Center, The Inter-Institute Center for Research on Marine Biodiversity, Resources and Biotechnologies, Fano, Italy

^3^Department of Materials, Environmental Sciences and Urban Planning, Polytechnic University of Marche, Via Brecce Bianche, 60131 Ancona, Italy

**Corresponding author*: Marco Candela, [marco.candela@unibo.it](mailto:marco.candela@unibo.it), Department of Pharmacy and Biotechnology, University of Bologna, Via Belmeloro 6, 40126 Bologna, Italy

**Supplementary material**

**
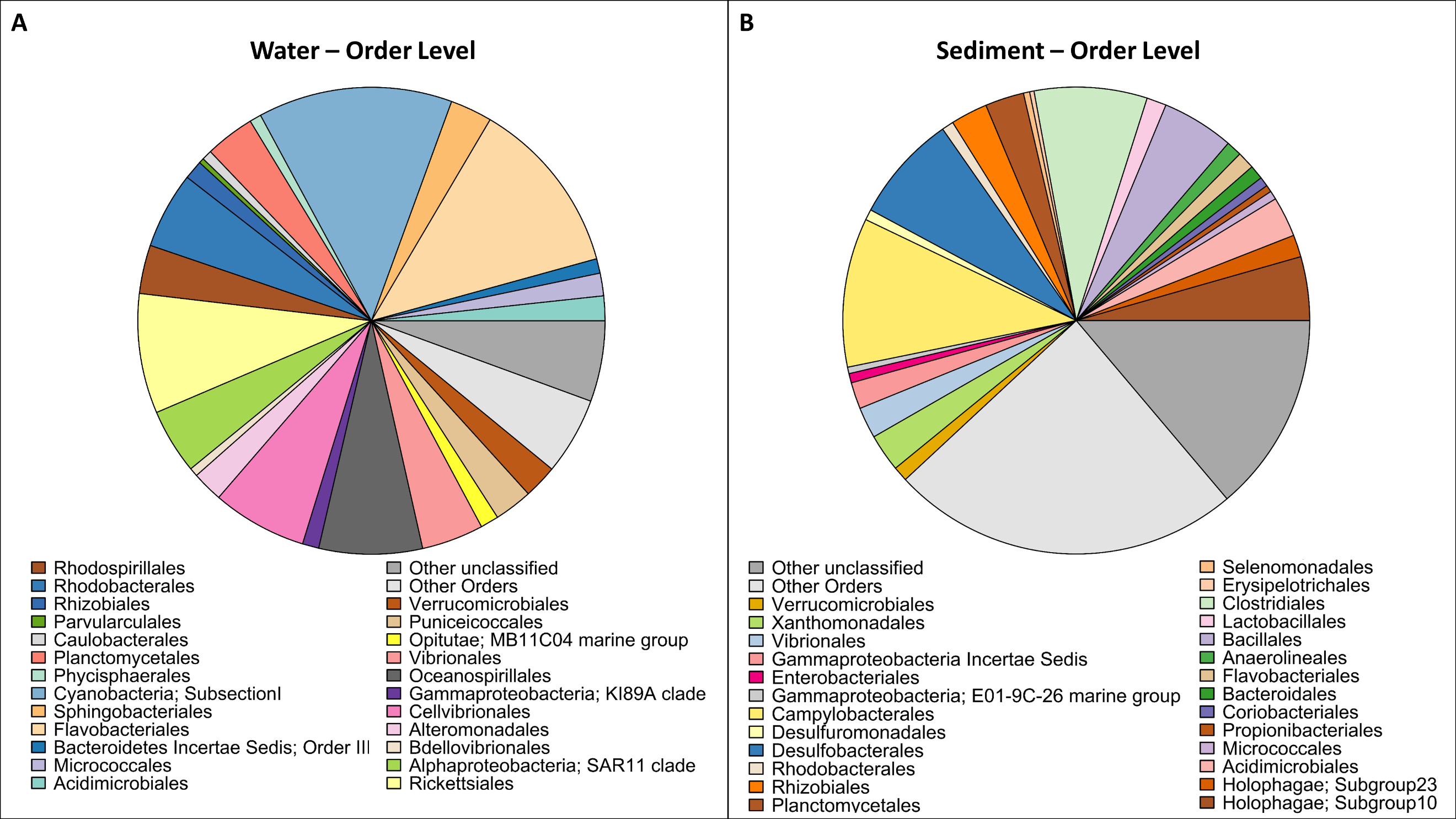
**

**Supplementary Figure 1:** Pie charts summarizing the compositional structure of the water (A) and sediment (B) microbiomes at order level. Only orders with relative abundance > 2.0% in at least 2 samples are represented.


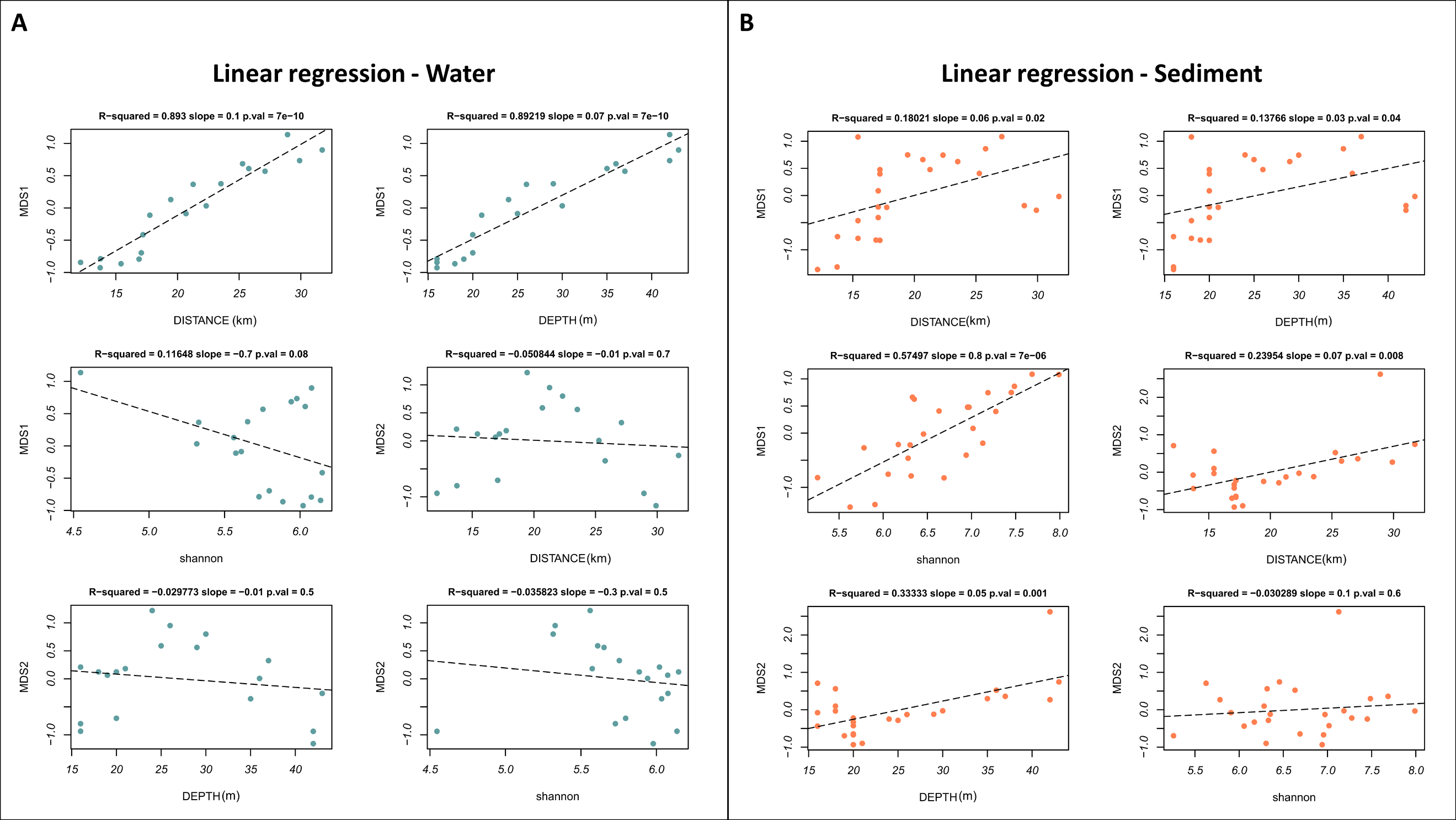


**Supplementary Figure 2:** Linear regression plots between the two axes (MDS1 and MDS2) of the PCoA of water (A) and sediment (B) samples and three parameters: Distance from coast, Depth of sampling point and Shannon index.


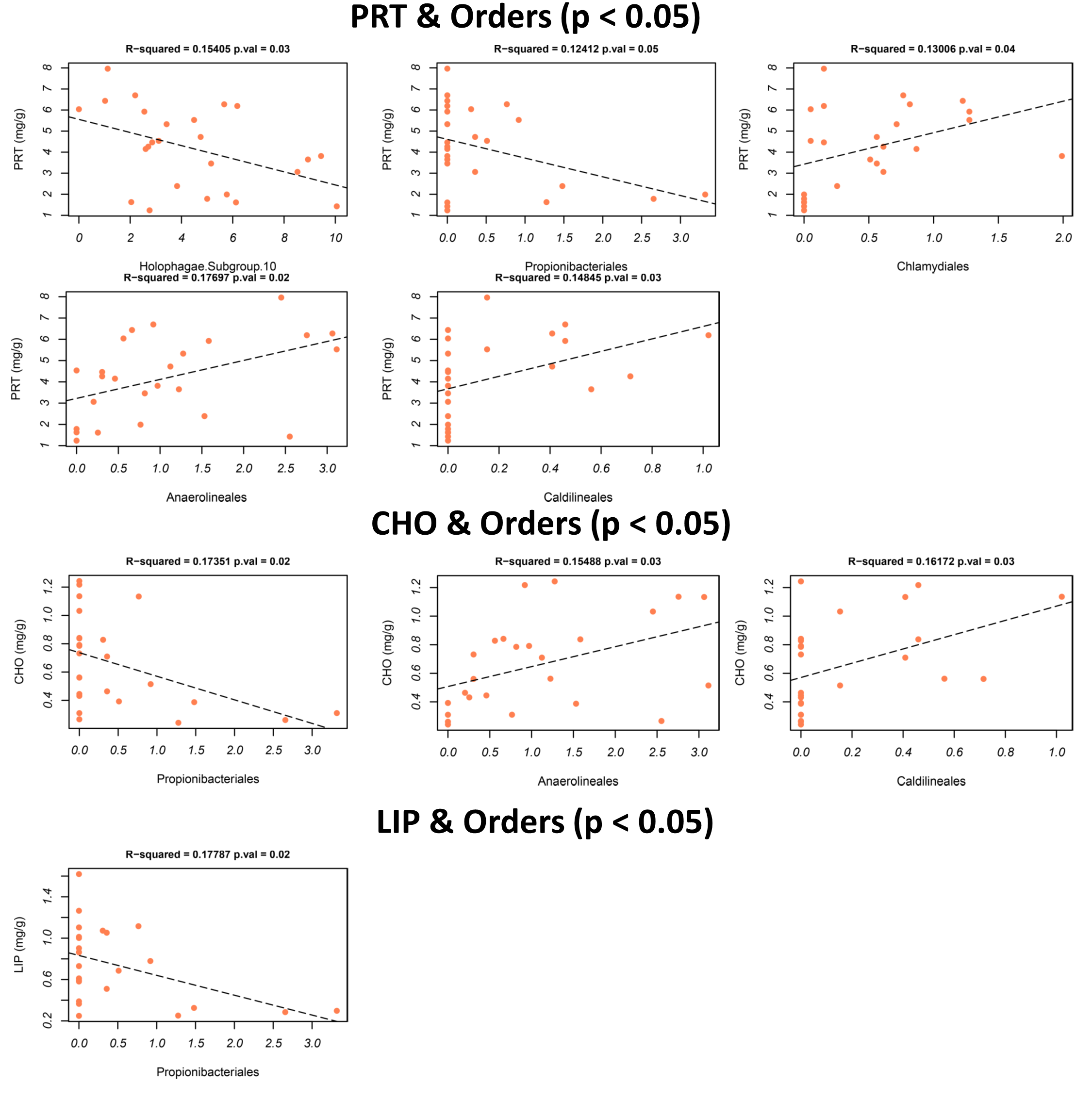


**Supplementary Figure 3:** Linear regression plots between bacterial Order abundance and biochemical components (PRT, CHO, LIP) in the sediment samples. Only relationship with a p < 0.05 are showed.


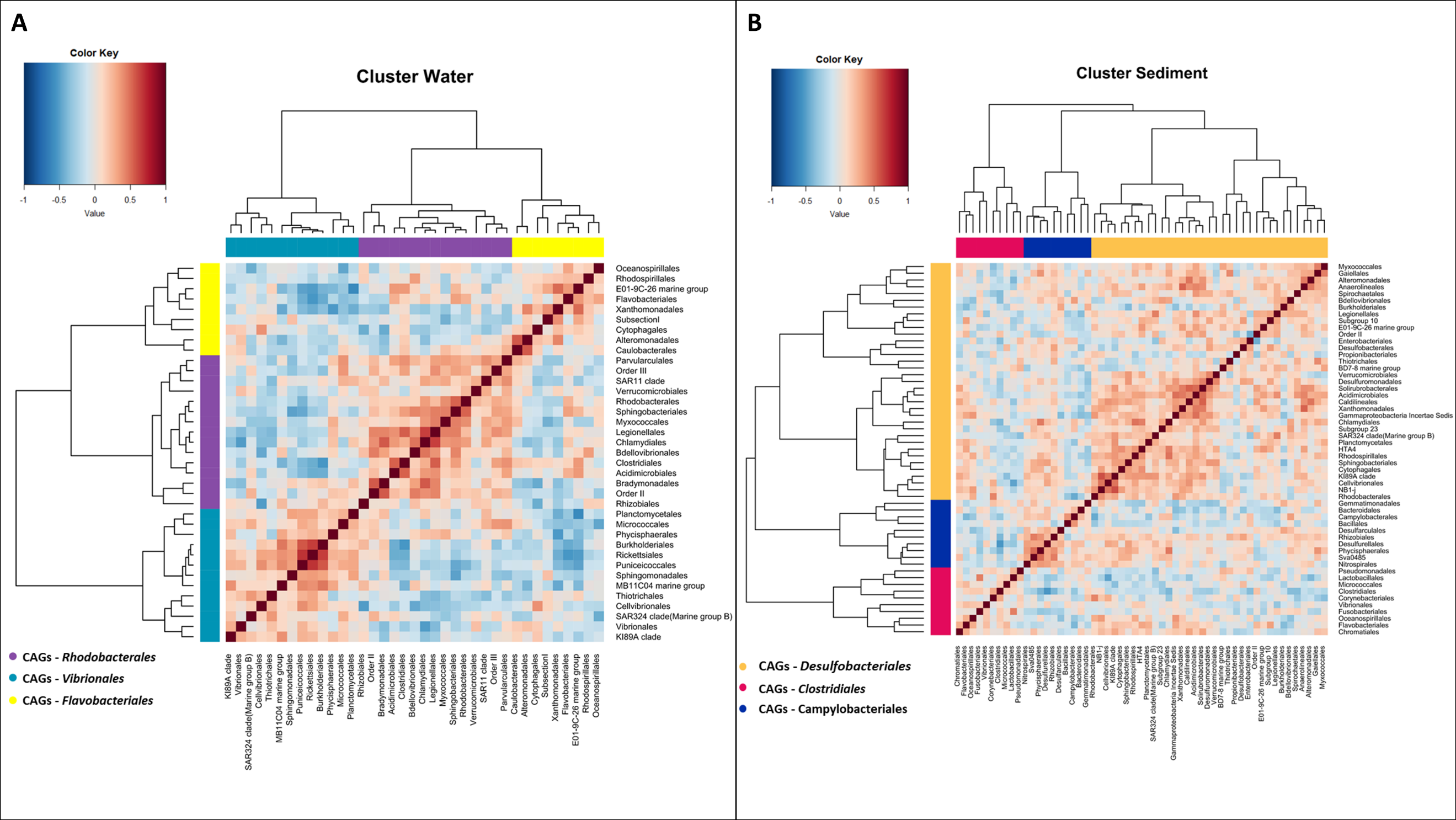


**Supplementary Figure 4:** Heatmaps of co-abundant groups (CAGs) identified by hierarchical clustering with the Spearman correlation. A) Heatmap for water microbiome at the order levels, B) Heatmap for sediment microbiome at the order levels. Colour scale for Spearman correlation is provided on the left of each panel, where red represents the highest and dark blue the lowest correlation. Bacterial orders abundance profile was clustered using hierarchical clustering approach, using the profile Spearman correlation and Ward’s linkage method. The obtained clusters were represented by dendrograms at the top and left margins of each Heatmap. Within each cluster, bacterial orders were coloured according to the colour legend in the bottom left of each panel.


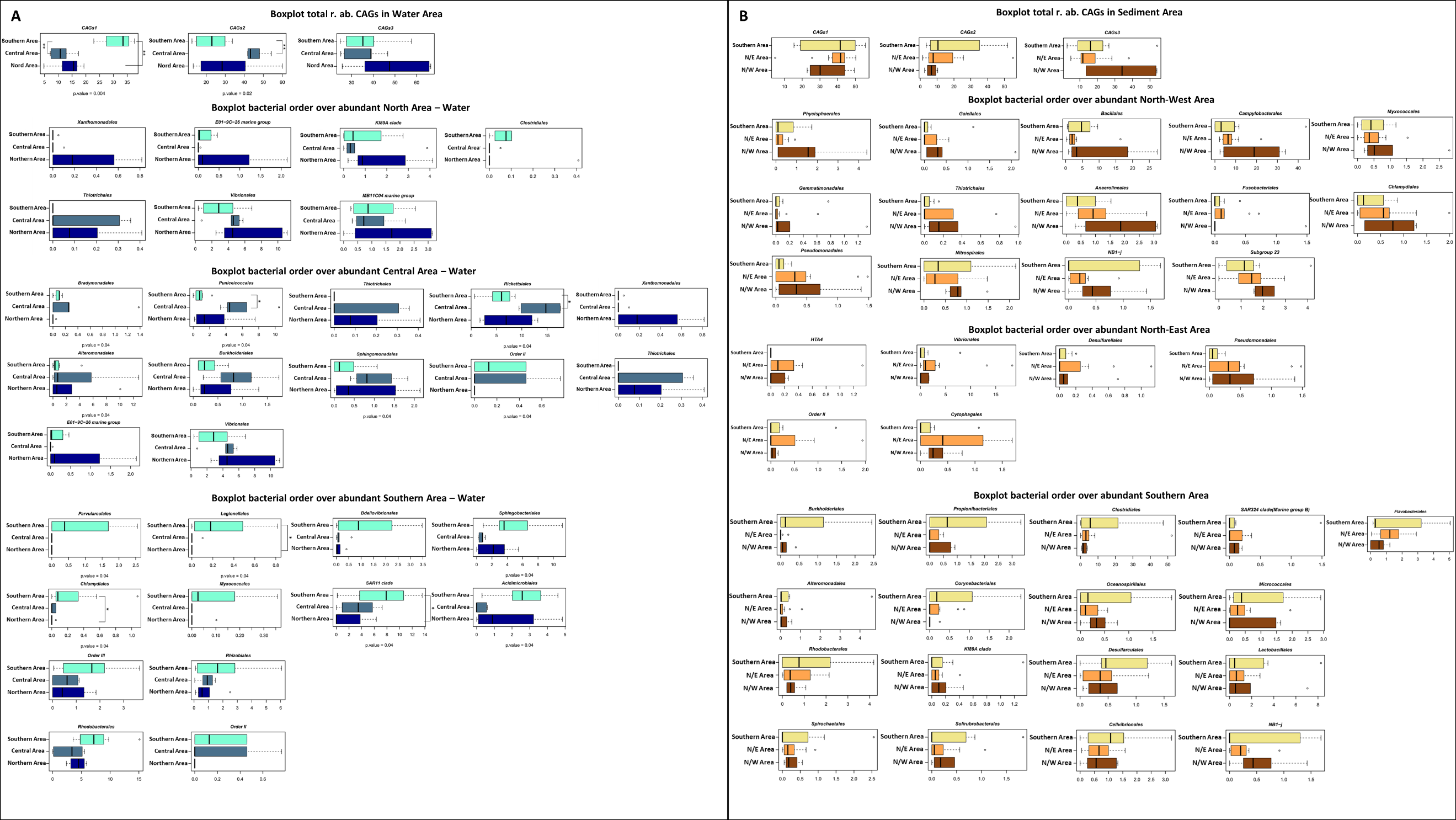


**Supplementary Figure 5:** Boxplots showing the variation of the relative abundance of CAGs (Upper part) and of each over abundant order in the same CAGs (over-abundance > 1.3, bottom part) across all sectors under study both for pelagic microbiome (Panel A) and sediment microbiome (Panel B). The central box of each dataset represents the distance between the 25th and the 75th percentiles. The median between them is marked with a black line. Significant variation across groups was highlighted in the figure (Wilcoxon rank-sum test; *p* ≤ 0.05 *, *p* ≤ 0.01 **).

**Supplementary Table 1:** Table reporting exact geographical coordinates of the 19 sampling sites with additional information of *distance from coast* (km) and *depth* (m).

|  | **DISTANCE FROM COAST (km)** | **DEPTH (m)** | **LATITUDE** | **LONGITUDE** |
| --- | --- | --- | --- | --- |
| Sampling Site 1 | 13.728 | 16 | 44.1346944 | 12.72288889 |
| Sampling Site 2 | 13.757 | 16 | 44.1060278 | 12.76027778 |
| Sampling Site 3 | 12.14 | 16 | 44.0686667 | 12.79630556 |
| Sampling Site 4 | 16.884 | 19 | 44.1516944 | 12.74511111 |
| Sampling Site 5 | 15.421 | 18 | 44.1311389 | 12.75394444 |
| Sampling Site 6 | 17.065 | 20 | 44.1075833 | 12.82425 |
| Sampling Site 6B | 17.202 | 20 | 44.1033889 | 12.83319444 |
| Sampling Site 7 | 17.757 | 21 | 44.1360278 | 12.78602778 |
| Sampling Site 8 | 20.688 | 25 | 44.1651111 | 12.79569444 |
| Sampling Site 9 | 21.269 | 26 | 44.1480556 | 12.83227778 |
| Sampling Site 10 | 19.454 | 24 | 44.1235833 | 12.84311111 |
| Sampling Site 11 | 23.521 | 29 | 44.1963333 | 12.81055556 |
| Sampling Site 12 | 25.78 | 35 | 44.1907778 | 12.85272222 |
| Sampling Site 13 | 25.275 | 36 | 44.1675278 | 12.88288889 |
| Sampling Site 14 | 22.325 | 30 | 44.1393611 | 12.87397222 |
| Sampling Site 15 | 27.099 | 37 | 44.2244444 | 12.83363889 |
| Sampling Site 16 | 31.735 | 43 | 44.2524444 | 12.87088861 |
| Sampling Site 17 | 29.905 | 42 | 44.2218889 | 12.88508333 |
| Sampling Site 18 | 28.924 | 42 | 44.1948611 | 12.90647222 |

| **Sediment Area** | | | **Water Area** | | |
| --- | --- | --- | --- | --- | --- |
| **Southern Sector** | | | **Southern Sector** | | |
| **Northeast Sector** | | | **Central Sector** | | |
| **Northwest Sector** | | | **Northern Sector** | | |
| **Samples** | **Shannon alpha-diversity** | **Quartile distribution** | **Samples** | **Shannon alpha-diversity** | **Quartile distribution** |
| **C4S** | 5.257365 | 1° | **C18W** | 4.546159 | 1° |
| **C3S** | 5.62557 | 1° | **C14W** | 5.316015 | 1° |
| **C17S** | 5.782988 | 1° | **C9W** | 5.329229 | 1° |
| **C1S** | 5.907133 | 1° | **C10W** | 5.561033 | 1° |
| **C2S** | 6.056522 | 1° | **C7W** | 5.574893 | 1° |
| **C6B2S** | 6.171907 | 1° | **C8W** | 5.611047 | 2° |
| **C5AS** | 6.282452 | 1° | **C11W** | 5.652874 | 2° |
| **C7S** | 6.304958 | 2° | **C2W** | 5.728202 | 2° |
| **C5CS** | 6.316561 | 2° | **C15W** | 5.754454 | 2° |
| **C8S** | 6.331336 | 2° | **C6BW** | 5.796217 | 3° |
| **C11S** | 6.351584 | 2° | **C5W** | 5.886421 | 3° |
| **C16S** | 6.456489 | 2° | **C13W** | 5.941774 | 3° |
| **C13S** | 6.632595 | 3° | **C17W** | 5.979078 | 3° |
| **C6BS** | 6.689232 | 3° | **C1W** | 6.020465 | 3° |
| **C6B1S** | 6.939868 | 3° | **C12W** | 6.034664 | 4° |
| **C6CS** | 6.955684 | 3° | **C4W** | 6.075191 | 4° |
| **C9S** | 6.971714 | 3° | **C16W** | 6.076636 | 4° |
| **C6AS** | 7.017395 | 3° | **C3W** | 6.136331 | 4° |
| **C18S** | 7.127725 | 4° | **C6W** | 6.147152 | 4° |
| **C14S** | 7.186221 | 4° |  |  |  |
| **C6B3S** | 7.27495 | 4° |  |  |  |
| **C10S** | 7.450581 | 4° |  |  |  |
| **C12S** | 7.48579 | 4° |  |  |  |
| **C15S** | 7.685893 | 4° |  |  |  |
| **C5BS** | 7.989378 | 4° |  |  |  |

**Supplementary Table 2**: Water and sediment samples quartile distribution according to the Shannon alpha-diversity values. Color legend indicate samples corresponding sector for both water and sediment area.

**Supplementary Table 3:** Concentrations of the different biochemical compounds of organic matter in the sediment samples of the investigated area. Data are reported as mean ± standard deviation. **PRT**: Total Protein; **CHO**: Total Carbohydrates; **LIP**: Total Lipids; **Chl-a**: Chlorophyll-A; **Pheo**: Pheopigments; **BPC**: Biopolymeric C.

| **Station** | **PRT**  **mg/g** | | **CHO**  **mg/g** | | **LIP**  **mg/g** | | **Chl-a**  **µg/g** | | **Pheo**  **µg/g** | | **BPC**  **mgC/g** | |
| --- | --- | --- | --- | --- | --- | --- | --- | --- | --- | --- | --- | --- |
|  | **Mean** | **Ds** | **Mean** | **Ds** | **Mean** | **Ds** | **Mean** | **Ds** | **Mean** | **Ds** | **Mean** | **Ds** |
| C1S | 1.99 | 0.47 | 0.31 | 0.07 | 0.30 | 0.09 | 0.70 | 0.07 | 8.96 | 0.15 | 1.32 | 0.17 |
| C2S | 2.39 | 0.22 | 0.39 | 0.02 | 0.33 | 0.04 | 0.94 | 0.04 | 10.20 | 0.30 | 1.57 | 0.14 |
| C3S | 1.24 | 0.23 | 0.31 | 0.04 | 0.25 | 0.11 | 0.59 | 0.12 | 8.27 | 1.05 | 0.92 | 0.15 |
| C4S | 4.15 | 1.46 | 0.45 | 0.03 | 0.36 | 0.08 | 0.69 | 0.01 | 7.08 | 0.65 | 2.49 | 1.04 |
| C5.1S | 1.78 | 0.06 | 0.26 | 0.01 | 0.28 | 0.02 | 0.63 | 0.11 | 9.65 | 0.31 | 1.19 | 0.03 |
| C5.2S | 4.26 | 0.62 | 0.56 | 0.02 | 0.73 | 0.02 | 2.14 | 0.04 | 24.08 | 1.49 | 2.85 | 0.32 |
| C5.3S | 1.63 | 0.12 | 0.24 | 0.01 | 0.25 | 0.06 | 0.57 | 0.05 | 6.49 | 0.58 | 1.08 | 0.08 |
| C6.1S | 3.06 | 1.55 | 0.46 | 0.30 | 0.51 | 0.11 | 1.40 | 0.19 | 14.90 | 2.40 | 2.07 | 0.80 |
| C6.2S | 1.43 | 0.09 | 0.27 | 0.01 | 0.39 | 0.04 | 1.23 | 0.20 | 14.49 | 1.46 | 1.08 | 0.08 |
| C6.3S | 1.61 | 0.15 | 0.43 | 0.11 | 0.58 | 0.20 | 1.59 | 0.61 | 18.29 | 4.48 | 1.29 | 0.02 |
| C6B.1S | 3.82 | 0.56 | 0.79 | 0.04 | 0.86 | 0.11 | 5.37 | 0.19 | 26.91 | 1.82 | 2.83 | 0.23 |
| C6B.2S | 4.54 | 0.16 | 0.39 | 0.10 | 0.69 | 0.11 | 0.57 | 0.08 | 10.97 | 0.97 | 2.89 | 0.13 |
| C6B.3S | 3.46 | 0.47 | 0.79 | 0.22 | 0.59 | 0.05 | 3.58 | 0.02 | 19.76 | 0.23 | 2.45 | 0.16 |
| C7S | 3.65 | 0.98 | 0.56 | 0.21 | 0.61 | 0.14 | 0.94 | 0.08 | 12.28 | 1.10 | 2.47 | 0.52 |
| C8S | 4.46 | 1.05 | 0.73 | 0.11 | 1.26 | 0.46 | 0.96 | 0.16 | 16.37 | 0.24 | 3.43 | 0.81 |
| C9S | 4.72 | 0.80 | 0.71 | 0.05 | 1.05 | 0.10 | 1.39 | 0.03 | 17.23 | 1.57 | 3.39 | 0.33 |
| C10S | 5.92 | 0.69 | 0.84 | 0.30 | 0.86 | 0.09 | 1.09 | 0.07 | 16.71 | 0.93 | 3.88 | 0.38 |
| C11S | 7.96 | 0.37 | 1.03 | 0.23 | 1.10 | 0.12 | 0.86 | 0.17 | 20.36 | 1.60 | 5.14 | 0.36 |
| C12S | 5.53 | 0.10 | 0.51 | 0.07 | 0.78 | 0.09 | 0.79 | 0.05 | 16.78 | 2.84 | 3.50 | 0.09 |
| C13S | 6.70 | 0.25 | 1.22 | 0.25 | 1.62 | 1.07 | 0.84 | 0.11 | 13.78 | 0.68 | 4.98 | 0.81 |
| C14S | 6.19 | 0.40 | 1.14 | 0.31 | 0.90 | 0.03 | 1.30 | 0.06 | 19.55 | 1.53 | 4.17 | 0.10 |
| C15S | 6.27 | 0.22 | 1.13 | 0.16 | 1.12 | 0.15 | 0.91 | 0.11 | 17.39 | 0.29 | 4.36 | 0.13 |
| C16S | 5.33 | 0.56 | 1.24 | 0.06 | 1.01 | 0.22 | 0.63 | 0.04 | 12.64 | 0.50 | 3.87 | 0.14 |
| C17S | 6.44 | 1.22 | 0.84 | 0.12 | 1.00 | 0.31 | 0.35 | 0.01 | 11.75 | 0.48 | 4.24 | 0.53 |
| C18S | 6.04 | 1.90 | 0.83 | 0.11 | 1.07 | 0.28 | 0.42 | 0.03 | 11.50 | 0.13 | 4.09 | 0.81 |

**Supplementary Table 4**: Bacterial orders CAGs component and mean relative abundance (%) in the 3 Areas for water and for sediment identified in the study. (3 pages)

| **Bacterial Orders** | **meanArea_N/W** | **meanArea_N/E** | **meanArea_Sud** | **CAG_sediment** |
| --- | --- | --- | --- | --- |
| *Holophagae;D_3_Subgroup 10* | 3.0968 | 5.281 | 4.205 | *Desulfobacterales* |
| *Holophagae;D_3_Subgroup 23* | 2.0248 | 1.4432 | 1.2825 | *Desulfobacterales* |
| *Acidimicrobiales* | 2.8416 | 3.0396 | 2.3928 | *Desulfobacterales* |
| *Propionibacteriales* | 0.2808 | 0.1392 | 1.0911 | *Desulfobacterales* |
| *Gaiellales* | 0.536 | 0.1439 | 0.1595 | *Desulfobacterales* |
| *Solirubrobacterales* | 0.2212 | 0.2135 | 0.402 | *Desulfobacterales* |
| *Bacteroidetes Incertae Sedis;D_3_Order II* | 0.051 | 0.3759 | 0.2169 | *Desulfobacterales* |
| *Cytophagales* | 0.2978 | 0.5894 | 0.1787 | *Desulfobacterales* |
| *Sphingobacteriales* | 0.3658 | 0.4873 | 0.4403 | *Desulfobacterales* |
| *Chlamydiales* | 0.7232 | 0.5476 | 0.2808 | *Desulfobacterales* |
| *Anaerolineales* | 1.8121 | 1.0673 | 0.536 | *Desulfobacterales* |
| *Caldilineales* | 0.1191 | 0.2135 | 0.1595 | *Desulfobacterales* |
| *Planctomycetales* | 2.782 | 2.4456 | 2.9543 | *Desulfobacterales* |
| *Rhodobacterales* | 0.4679 | 0.6914 | 1.2634 | *Desulfobacterales* |
| *Rhodospirillales* | 0.4849 | 0.5058 | 0.6381 | *Desulfobacterales* |
| *Burkholderiales* | 0.1106 | 0.0232 | 0.6253 | *Desulfobacterales* |
| *Bdellovibrionales* | 0.604 | 0.5058 | 0.6891 | *Desulfobacterales* |
| *Desulfobacterales* | 5.7342 | 8.5433 | 7.2231 | *Desulfobacterales* |
| *Desulfuromonadales* | 0.604 | 0.7703 | 0.7976 | *Desulfobacterales* |
| *Myxococcales* | 0.9018 | 0.4826 | 0.4786 | *Desulfobacterales* |
| *Deltaproteobacteria;D_3_NB1-j* | 0.553 | 0.2274 | 0.536 | *Desulfobacterales* |
| *SAR324 clade(Marine group B)* | 0.0851 | 0.0975 | 0.2042 | *Desulfobacterales* |
| *Alteromonadales* | 0.1531 | 0.1578 | 0.6764 | *Desulfobacterales* |
| *Gammaproteobacteria;D_3_BD7-8 marine group* | 0.3403 | 0.4362 | 0.4148 | *Desulfobacterales* |
| *Cellvibrionales* | 0.6636 | 0.71 | 1.123 | *Desulfobacterales* |
| *Gammaproteobacteria;D_3_E01-9C-26 marine group* | 0.502 | 0.5244 | 0.3063 | *Desulfobacterales* |
| *Enterobacteriales* | 0.6721 | 0.6358 | 0.6445 | *Desulfobacterales* |
| *Gammaproteobacteria Incertae Sedis* | 1.3187 | 2.311 | 1.5761 | *Desulfobacterales* |
| *Gammaproteobacteria;D_3_HTA4* | 0.0766 | 0.2552 | 0 | *Desulfobacterales* |
| *Gammaproteobacteria;D_3_KI89A clade* | 0.1446 | 0.0789 | 0.2042 | *Desulfobacterales* |
| *Legionellales* | 0.6126 | 0.7425 | 0.7912 | *Desulfobacterales* |
| *Thiotrichales* | 0.2808 | 0.1671 | 0.0319 | *Desulfobacterales* |
| *Xanthomonadales* | 3.335 | 2.3899 | 2.3418 | *Desulfobacterales* |
| *Spirochaetales* | 0.2467 | 0.2552 | 0.4977 | *Desulfobacterales* |
| *Verrucomicrobiales* | 0.4509 | 1.2901 | 1.0975 | *Desulfobacterales* |
| **Bacterial Orders** | **meanArea_N/W** | **meanArea_N/E** | **meanArea_Sud** | **CAG_sediment** |
| *Corynebacteriales* | 0.0425 | 0.1949 | 0.5998 | *Clostridiales* |
| *Micrococcales* | 0.519 | 0.4037 | 0.9188 | *Clostridiales* |
| *Flavobacteriales* | 0.519 | 1.2994 | 1.5888 | *Clostridiales* |
| *Lactobacillales* | 1.6675 | 0.7935 | 1.9206 | *Clostridiales* |
| *Clostridiales* | 1.9993 | 7.3693 | 12.7808 | *Clostridiales* |
| *Fusobacteriales* | 0.2467 | 0.1624 | 0.0702 | *Clostridiales* |
| *Chromatiales* | 0.2297 | 0.1299 | 0.2106 | *Clostridiales* |
| *Oceanospirillales* | 0.3573 | 0.1995 | 0.536 | *Clostridiales* |
| *Pseudomonadales* | 0.4679 | 0.4223 | 0.0766 | *Clostridiales* |
| *Vibrionales* | 0.553 | 3.7496 | 1.1741 | *Clostridiales* |
| **Bacterial Orders** | **meanArea_N/W** | **meanArea_N/E** | **meanArea_Sud** | **CAG_sediment** |
| *Bacteroidales* | 0.4594 | 1.2019 | 1.0528 | *Campylobacteriales* |
| *Bacillales* | 9.1969 | 3.086 | 4.4921 | *Campylobacteriales* |
| *Gemmatimonadales* | 0.2637 | 0.0742 | 0.1085 | *Campylobacteriales* |
| *Nitrospirales* | 0.8423 | 0.4501 | 0.6253 | *Campylobacteriales* |
| *Phycisphaerales* | 1.5995 | 0.2227 | 0.453 | *Campylobacteriales* |
| *Rhizobiales* | 2.4758 | 2.4038 | 2.8139 | *Campylobacteriales* |
| *Desulfarculales* | 0.3743 | 0.3944 | 0.7147 | *Campylobacteriales* |
| *Desulfurellales* | 0.1531 | 0.2135 | 0.0447 | *Campylobacteriales* |
| *Deltaproteobacteria;D_3_Sva0485* | 0.2042 | 0.2227 | 0.2425 | *Campylobacteriales* |
| *Campylobacterales* | 18.2066 | 7.3043 | 8.5567 | *Campylobacteriales* |
| **Bacterial Orders** | **meanArea_N** | **meanArea_C** | **meanArea_S** | **CAG_water** |
| *Acidimicrobiales* | 1.659 | 0.2348 | 2.6672 | *Rhodobacterales* |
| *Bacteroidetes Incertae Sedis;D_3_Order II* | 0 | 0.245 | 0.2042 | *Rhodobacterales* |
| *Bacteroidetes Incertae Sedis;D_3_Order III* | 0.6636 | 0.5615 | 1.5442 | *Rhodobacterales* |
| *Sphingobacteriales* | 2.2716 | 0.633 | 4.7984 | *Rhodobacterales* |
| *Chlamydiales* | 0.0085 | 0.0204 | 0.2489 | *Rhodobacterales* |
| *Clostridiales* | 0.0681 | 0.0102 | 0.0638 | *Rhodobacterales* |
| *Parvularculales* | 0 | 0 | 0.8359 | *Rhodobacterales* |
| *Rhizobiales* | 0.8508 | 0.8678 | 1.9461 | *Rhodobacterales* |
| *Rhodobacterales* | 4.3389 | 2.8484 | 7.5485 | *Rhodobacterales* |
| *Alphaproteobacteria;D_3_SAR11 clade* | 1.6845 | 3.4712 | 7.2677 | *Rhodobacterales* |
| *Bdellovibrionales* | 0.1021 | 0.1736 | 1.2379 | *Rhodobacterales* |
| *Bradymonadales* | 0.0085 | 0.3777 | 0.083 | *Rhodobacterales* |
| *Myxococcales* | 0.017 | 0 | 0.0957 | *Rhodobacterales* |
| *Legionellales* | 0 | 0.0204 | 0.2744 | *Rhodobacterales* |
| *Verrucomicrobiales* | 2.229 | 1.6029 | 2.8586 | *Rhodobacterales* |
| **Bacterial Orders** | **meanArea_N** | **meanArea_C** | **meanArea_S** | **CAG_water** |
| *Micrococcales* | 0.6551 | 2.3379 | 1.7675 | *Vibrionales* |
| *Phycisphaerales* | 0.8423 | 1.2047 | 0.5296 | *Vibrionales* |
| *Planctomycetales* | 1.4974 | 5.5641 | 3.535 | *Vibrionales* |
| *Rickettsiales* | 7.2316 | 13.8438 | 5.6279 | *Vibrionales* |
| *Sphingomonadales* | 0.7402 | 1.0107 | 0.2871 | *Vibrionales* |
| *Burkholderiales* | 0.4509 | 0.8984 | 0.2935 | *Vibrionales* |
| *Deltaproteobacteria;D_3_SAR324 clade(Marine group B)* | 0.1787 | 0.3573 | 0.3892 | *Vibrionales* |
| *Cellvibrionales* | 7.7335 | 8.5248 | 4.4921 | *Vibrionales* |
| *Gammaproteobacteria;D_3_KI89A clade* | 1.5995 | 0.9699 | 0.8933 | *Vibrionales* |
| *Thiotrichales* | 0.1276 | 0.1327 | 0 | *Vibrionales* |
| *Vibrionales* | 6.1171 | 4.1552 | 2.9926 | *Vibrionales* |
| *Opitutae;D_3_MB11C04 marine group* | 1.6845 | 1.0209 | 1.0975 | *Vibrionales* |
| *Puniceicoccales* | 2.4332 | 5.7887 | 0.8359 | *Vibrionales* |
| **Bacterial Orders** | **meanArea_N** | **meanArea_C** | **meanArea_S** | **CAG_water** |
| *Cytophagales* | 0.3999 | 0.3369 | 0.2425 | *Flavobacteriales* |
| *Flavobacteriales* | 15.2118 | 7.4834 | 12.9786 | *Flavobacteriales* |
| *SubsectionI* | 16.4284 | 12.8739 | 11.5748 | *Flavobacteriales* |
| *Caulobacterales* | 0.5105 | 0.8576 | 0.7338 | *Flavobacteriales* |
| *Rhodospirillales* | 3.3095 | 3.4507 | 3.3435 | *Flavobacteriales* |
| *Alteromonadales* | 2.3396 | 3.8897 | 0.8742 | *Flavobacteriales* |
| *Gammaproteobacteria;D_3_E01-9C-26 marine group* | 0.5955 | 0.0102 | 0.1404 | *Flavobacteriales* |
| *Oceanospirillales* | 9.0948 | 6.3502 | 6.1958 | *Flavobacteriales* |
| *Xanthomonadales* | 0.2893 | 0.0204 | 0.0064 | *Flavobacteriales* |
